# Overexpression of the ribosome-inactivating protein *OsRIP1* modulates the jasmonate signaling pathway in rice

**DOI:** 10.3389/fpls.2024.1385477

**Published:** 2024-08-14

**Authors:** Simin Chen, Noémie De Zutter, Anikó Meijer, Koen Gistelinck, Pieter Wytynck, Isabel Verbeke, Vinicius J. S. Osterne, Subramanyam Kondeti, Tim De Meyer, Kris Audenaert, Els J. M. Van Damme

**Affiliations:** ^1^ Department of Biotechnology, Faculty of Bioscience Engineering, Ghent University, Ghent, Belgium; ^2^ Laboratory of Applied Mycology and Phenomics, Department of Plants and Crops, Faculty of Bioscience Engineering, Ghent University, Ghent, Belgium; ^3^ Department of Data Analysis & Mathematical Modelling, Ghent University, Ghent, Belgium

**Keywords:** ribosome-inactivating protein, *Oryza sativa*, *OsRIP1*, jasmonate signaling, photosynthesis

## Abstract

Ribosome-inactivating proteins (RIPs) are plant enzymes that target the rRNA. The cytoplasmic RIP, called OsRIP1, plays a crucial role in regulating jasmonate, a key plant hormone. Understanding the role of OsRIP1 can provide insights into enhancing stress tolerance and optimizing growth of rice. Transcription profiling by mRNA sequencing was employed to measure the changes in gene expression in rice plants in response to MeJA treatment. Compared to wild type (WT) plants, *OsRIP1* overexpressing rice plants showed a lower increase in mRNA transcripts for genes related to jasmonate responses when exposed to MeJA treatment for 3 h. After 24 h of MeJA exposure, the mRNA transcripts associated with the gibberellin pathway occurred in lower levels in *OsRIP1* overexpressing plants compared to WT plants. We hypothesize that the mechanism underlying OsRIP1 antagonization of MeJA-induced shoot growth inhibition involves cytokinin-mediated leaf senescence and positive regulation of cell cycle processes, probably via OsRIP1 interaction with 40S ribosomal protein S5 and α-tubulin. Moreover, the photosystem II 10kDa polypeptide was identified to favorably bind to OsRIP1, and its involvement may be attributed to the reduction of photosynthesis in *OsRIP1*-overexpressing plants subjected to MeJA at the early timepoint (3 h).

## 1 Introduction

Plants possess a sophisticated innate immune system to adapt to ever-changing environmental conditions. Jasmonates (JAs), salicylic acid and ethylene are the archetypal hormones and play critical roles in plant defense signaling (Pieterse et al., 2009; De Vleesschauwer et al., 2013). Other hormones such as gibberellins (GAs), abscisic acid, cytokinins and auxins have emerged as important regulators not only in plant growth and development, but also in plant immunity by interfering with the salicylic acid-jasmonate/ethylene backbone of plant basal immunity. The complicated network of communication among different plant hormone signaling pathways is often referred to as the hormone crosstalk in plant disease and defense (Robert-Seilaniantz et al., 2011; Klessig et al., 2018). JA, a lipid-derived plant hormone, functions as a core signal in plant developmental processes and in responses to abiotic stress and biotic stresses (Kazan and Manners, 2011; Yang et al., 2019). Different JA compounds are synthesized in the cytoplasm, such as methyl jasmonate (MeJA), JA–isoleucine and 12-hydroxyjasmonic acid (Wasternack and Strnad, 2016). These JAs are known to modulate plant defense upon insect herbivory and pathogen infection (Li et al., 2022).

The cell nucleus has attracted attention as a new source of yet unknown molecules involved in various signaling pathways of plant defense responses (Kalinina et al., 2018). Its primary function is ribosomal RNA (rRNA) synthesis and ribosome biogenesis. Ribosome-inactivating proteins (RIPs) with rRNA N-glycosylase activity target the 23S/25S/28S rRNAs, leading to irreversible inhibition of translation. A large number of studies indicate that plant RIPs play important roles in defense against pathogens and insects, which may partly be attributed to crosstalk mechanisms between these proteins and phytohormones or reactive oxygen species (ROS) (Zhu et al., 2018). The RIP family consists of 2 groups of proteins, type 1 RIPs which are single-chained proteins and type 2 RIPs composed of a type 1 RIP chain linked to a lectin chain. We previously reported that transgenic rice seedlings overexpressing *OsRIP1* (LOC_Os01g06740) exhibited reduced susceptibility to exogenous MeJA application (Wytynck et al., 2021). In addition, OsRIP1 has been reported as a JA-inducible protein (Kawahara et al., 2016), and emerging evidence reveals that transgenic rice plants targeting JA-related genes showed altered expression of the OsRIP1 gene, implying the intricate relationship between OsRIP1 and JA signaling. In the background of *Japonica* rice (*Oryza sativa* L.) variety Zhonghua 11, leaf expression of OsRIP1 was up-regulated significantly in OsBAG4-overexpressing rice plants (fold-change 4.41) (**Supplementary Materials 1**, **Supplementary Figure S1**) and in the ebr1 mutant (fold-change 2.01) (**Supplementary Figure S2**). EBR1 encodes an RING-type E3 ligase that interacts with OsBAG4, a Bcl-2-associated athanogene protein, leading to its ubiquitination and degradation (Kabbage and Dickman, 2008). *OsBAG4*-overexpressing rice plants and the mutant ebr1 were characterized by the activation of many defense-related genes involved in the JA and salicylic acid pathways, and enhanced resistance to bacterial blight caused by *Xanthomonas oryzae* pv. *oryzae* (*Xoo*) and fungal blast caused by *Magnaporthe oryzae* (You et al., 2016). OsJAZ1 (LOC_Os04g55920.1) has a negative effect on drought tolerance, and *OsJAZ1*-overexpressing plants under drought stress showed different expression levels for abscisic acid and the JA signaling as well as stress-responsive genes, including the up-regulation of *OsRIP1* (fold-change 3.15) (**Supplementary Materials 1**, **Supplementary Figure S3**) but the repression of both *OsbHLH006* and *OsbHLH148* (Fu et al., 2017). The latter two genes are both basic helix-loop-helix proteins and confer drought tolerance in rice through interacting with OsJAZ proteins in the JA signaling pathway (Seo et al., 2011; Miyamoto et al., 2013). Notably, *OsRIP1* and *OsJAZ12* transcripts were dramatically enhanced up to 26.22-fold (**Supplementary Materials 1**, **Supplementary Figure S4**) and 34.51-fold (**Supplementary Figure S5**) in the JA overproduction mutant, *cea62*, and in this mutant the JA pathway was activated by depletion of the hydroperoxide lyase OsHL3 (LOC_Os02g02000) from the CYP74B subfamily of the cytochrome P450 family, leading to resistance to the *Xoo* T1 strain (Liu et al., 2012). These observations all support the involvement of OsRIP1 in the JA signaling pathway in rice. In the present study, we focus on an in-depth investigation of OsRIP1 and JA-mediated pathways via transcriptome profiling analysis. Previously, transgenic rice plants from line H were demonstrated to exhibit 31-fold and 65-fold higher expression of OsRIP1 in shoots and roots, respectively, while in the case from line J this was 21-fold and 42-fold (Wytynck, 2020). In this study, we provide the first insight into how OsRIP1 antagonizes MeJA-induced leaf senescence using transgenic *OsRIP1*-overexpressing plants subjected to exogenous MeJA application. In addition, health parameters of plants and interaction partners for OsRIP1 were studied to uncover the physiological mechanisms underlying OsRIP1 functions in rice.

## 2 Materials and methods

### 2.1 Plant materials and seed sterilization

Seeds of wild type (WT) rice *Oryza sativa* cv. *Nipponbare* and *OsRIP1*-OE plants (T4 generation) from line J and line H (Wytynck et al., 2021) were dehusked, and soaked in 70% ethanol on a shaker (130 rpm) for 5 minutes, followed by 45 minutes washing in 5% NaOCl solution containing 0.01% Tween-20. After extensive washing with sterilized H_2_O seeds were incubated overnight in H_2_O on a shaker (130 rpm) at 28°C.

### 2.2 Phenotypic analysis of transgenic rice

Sterilized seeds were germinated in ½ solid MS medium (pH 5.8) supplemented with 30 g/l sucrose, 8 g/l Agarose SPI (Duchefa Biochemie, Netherlands) and 1.12 mg/l Gamborg B5 vitamins (Duchefa) in square Petri dishes, sealed with micropore tape. Seeds of transgenic plants from line J and line H were germinated on selective medium containing 4 mg/l phosphinothricin (Duchefa). Petri dishes were wrapped with aluminum foil and incubated in the dark in a plant chamber at 28°C. After 4 days, the aluminum foil was removed, and germinated seeds were grown at 28°C with a 16-h light/8-h dark cycle for an additional 3 days. One-week-old rice seedlings were grown hydroponically in the ½ Hoagland solution under the same conditions in a plant cabinet. The ½ Hoagland solution was refreshed daily. 14-day-old plants were photographed and the phenotypic differences between WT and *OsRIP1*-OE transgenic plants were analyzed for biomass, shoot length and root length.

### 2.3 MeJA treatment

14-day-old plants were subjected to MeJA treatment. MeJA (Sigma-Aldrich, Darmstadt, Germany) was dissolved in absolute ethanol (Sigma-Aldrich) to obtain a 100 mM stock solution, and then added to the ½ Hoagland solution to reach the working concentration of 100 μM. Control seedlings were kept in the ½ Hoagland solution with 0.1% (v/v) ethanol. Shoots and roots were sampled at 3 h and 24 h, and stored at -80°C. All treatments were set up for biological triplicates.

### 2.4 mRNA sequencing

Four treatment groups were set up for each indicated timepoint (3 h and 48 h), namely mock-treated WT plants, MeJA-treated WT plants, mock-treated T4 *OsRIP1*-OE transgenic rice plants line J, and MeJA-treated T4 *OsRIP1*-OE transgenic rice plants line J. Three independent biological replicates were performed for each treatment, containing 10-12 individual plants per replicate. Total RNA was extracted from freshly ground material using the Spectrum Plant Total RNA kit (Sigma-Aldrich). Library preparation, sequencing and data analysis were performed as previously described (Ghaemi et al., 2020). For each time point, Gene Ontology (GO) enrichment analyses were performed for significantly differentially expressed genes (Adjusted P-values, FDR< 0.05, and |log_2_-FC| > 1) using PLAZA 4.5 (https://bioinformatics.psb.ugent.be/plaza/versions/plaza_v4_5_monocots/).

### 2.5 Transcript analysis by quantitative RT-PCR

The reverse-transcribed cDNA was synthesized using Maxima First-Strand Synthesis kit (Thermo Fisher Scientific, Waltham, Massachusetts, USA) after RNA extraction and DNAse treatment. Transcript levels for genes of interest were analyzed by reverse transcription quantitative polymerase chain reaction (RT-qPCR) using gene specific primers (**Supplementary materials 1**, **Supplementary Table S1**). EXP, EXPNar and EIF5C were selected as the reference genes. RT-qPCR was performed using the 96-well CFX Connect™ Real-Time PCR Detection System (BioRad, Hercules, California, U.S.) with iQ™ SYBR^®^ Green Supermix (BioRad). The PCR amplification steps were 95°C for 10 min, followed by 41 cycles of 95°C for 15 sec, 60°C for 25 sec, 72°C for 20 sec. Three biological replicates with technical duplicates were performed for the RT-qPCR.

### 2.6 Evaluation of health parameters by multispectral imaging

After treatment with 100 μM MeJA, 14-day-old rice plants of WT, *OsRIP1*-OE line J and line H were cultured in a plant chamber at 28°C with 16 h light: 8 h dark. Plant health development was monitored longitudinally (0, 3, 6, 9, 24 and 48 h) through multispectral imaging analysis, including efficiency of photosystem II (Fv/Fm) (Baker, 2008), chlorophyll index (Chlldx) (Gitelson et al., 2003), modified anthocyanin reflectance index (mARI) (Gitelson et al., 2009) and biomass approximation based on the number of pixels occupied by the plant. At each timepoint, side-view images were captured to provide a more precise view of rice plants and leaves (n = 15 plants, 3 plants/image). At each timepoint 15 plants were used for the side-view images and discarded after the image acquisition. All the images were captured by a custom-build multispectral imaging- and microdispenser platform, equipped with WIWAM system and 6-Mp 16-bit 3CCD top-viewer camera (PhenoVation B.V., Wageningen, The Netherlands). Data processing was performed using the “Data Analysis Software” program (PhenoVation B.V.). Additionally, the phenotypic differences between WT and *OsRIP1*-OE transgenic plants were analyzed for biomass, shoot length and root length after 48 h of mock treatment or MeJA treatment, respectively.

### 2.7 Protein extraction from rice shoots

Frozen shoot samples from WT plants subjected to 100 μM MeJA treatment were crushed in the presence of liquid nitrogen. The powder was homogenized in ice-cold extraction buffer containing 25 mM Tris-HCl, 15 mM MgCl_2_, 150 mM NaCl, 0.1% NP-40, 1 mM PMSF, 1 μM E64 and 0.1% benzonase (pH 7.6) in a ratio of 2:1 (2 ml of buffer per gram of plant material). Samples were vortexed for 30 seconds followed by 30 seconds cooling on ice for a total of 10 minutes, followed by incubation on a rotary shaker for 30 minutes. The supernatant was recovered by centrifugation at 14,000 rpm for 20 min at 4°C and used as the protein extracts for the pull-down assays. All steps were performed on ice. Protein content of the extracts was determined using the Bradford method (Bradford, 1976).

### 2.8 Recombinant production and purification of recombinant OsRIP1

Recombinant OsRIP1 was produced in *E. coli* strain Rosetta (DE3) grown at 14°C for 72 h, and purified as described previously (De Zaeytijd et al., 2019).

### 2.9 Pull-down assays

Pull-down assays were performed using purified recombinant OsRIP1 as bait and protein extracts from shoots of MeJA-treated WT plants as prey. Briefly, 100 μg total rice protein was supplemented with approximately 54 μg purified OsRIP1, and incubated for 30 min at 4°C. 25 μl of Ni-NTA agarose beads (Qiagen, Hilden, Germany) were equilibrated with binding buffer (mix of the phosphate buffer from OsRIP1 purification and plant protein extraction buffer in a ratio of 8.3:1). The beads were incubated with the mix of protein extracts and OsRIP1 for 30 min at 4°C. After centrifugation, the beads were washed once with the binding buffer containing 50 mM imidazole followed by three washes with trypsin digest buffer (20 mM Tris-HCl, 2 mM CaCl_2_, pH 8.0). The beads were resuspended in 150 µl trypsin digest buffer and stored at -20°C prior to LC-MS/MS analysis. All incubations were performed on ice. Ni-NTA beads incubated with plant protein extracts only were used as a control. Four biological replicates were performed in total, one replicate was used for silver staining and Western blot analysis, and the other three replicates were used for LC-MS/MS analysis.

### 2.10 SDS-PAGE, silver staining, and Western blot analysis

Proteins were separated by SDS-PAGE using 15% acrylamide gels (Laemmli, 1970), followed by visualization with Pierce™ Silver Stain Kit (Thermo Scientific, Waltham, MA, United States). For Western blot analysis proteins were transferred from the acrylamide gel to polyvinylidene fluoride transfer membranes (FluoroTrans^®^ PVDF, Pall Laboratory, USA). Membranes were blocked in Tris-buffered saline (TBS: 10 mM Tris, 150 mM NaCl, 0.1% (v/v) Triton X-100, pH 7.6) containing 5% (w/v) non-fat milk powder. Subsequently, membranes were incubated with the primary anti-His antibody (1:1000, Thermo Fisher Scientific) for 1 h. Membranes were washed three times with TBS prior to incubation with rabbit anti-mouse IgG secondary antibody labelled with horseradish peroxidase (1:10,000, Dako, Glostrup, Denmark) for another hour. Following two washes with TBS and one wash with 0.1 M Tris buffer (pH 7.6), blots were detected and visualized using 0.025% (w/v) 3,3’-diaminobenzidine tetrahydrochloride (Sigma-Aldrich) containing 0.003% (v/v) hydrogen peroxide.

### 2.11 LC-MS/MS analysis

All proteins were separated from Ni-NTA beads by trypsin digest. After acidification and purification, purified peptides were determined by LC-MS/MS analysis using an Ultimate 3000 RSLC nano LC (Thermo Fisher Scientific, Bremen, Germany) in-line connected to a Q Exactive mass spectrometer (Thermo Fisher Scientific) equipped with a pneuNimbus dual ion source (Phoenix S&T). LC-MS/MS runs were searched using the MaxQuant algorithm (version 1.6.3.4) with mainly default search settings, including a false discovery rate set at 1% on both the peptide and protein level. Spectra were searched against the OsRIP1-6x His sequence and *Oryza sativa* proteins in the Uniprot database (database release version of March 2021 containing 39,947 protein sequences) (www.uniprot.org). Only proteins with at least one unique or razor peptide were retained leading to the identification of 1,443 proteins. Proteins were quantified by the MaxLFQ algorithm integrated in the MaxQuant software. A minimum ratio count of two unique or razor peptides was required for quantification. Further data analysis was performed with the Perseus software (version 1.6.2.1) after loading the protein groups file from MaxQuant.

Reverse database hits were removed, and replicate samples were grouped. Proteins with less than three valid values in at least one group were removed and missing values were imputed from a normal distribution centered around the detection limit leading to a list of 950 quantified proteins that was used for further data analysis. To compare protein abundance between OsRIP1-treated and OsRIP1-nontreated (CTRL) samples, statistical testing for differences between these two groups was performed, using the package limma. Statistical significance for differential regulation was set at FDR = 0.05 and |log_2_FC| = 1, and a volcano plot was also generated. The proteins shown to be differentially abundant between groups were visualized in a heatmap after non-supervised hierarchical clustering of z-scored protein LFQ intensities. The proteomics analyses were performed by the VIB Proteomics Core, Center for Medical Biotechnology, UGent Department of Biomolecular Medicine. Mass spectrometry was performed in triplicates.

### 2.12 Protein-protein docking

Protein-protein docking between OsRIP1 and the proteins identified in the pulldown assays has been performed using ClusPro 2.0 (Kozakov et al., 2017) with standard settings. Protein structure files in.pdb format have been obtained using AlphaFold2 (Jumper et al., 2021) using their respective Uniprot codes (**Table 1**) and selected based on model quality, with low-quality models being removed from further analysis. Simulations followed steps including rigid-body docking through the fast Fourier transform approach, root-mean square deviation clustering of structures, and refinement of the structures on the representative clusters. Poses were selected based on the most overrepresented clusters.

**Table 1:** Potential interaction partners of OsRIP1 as identified by LC-MS/MS after pull-down assays.

Log_2_ (OsRIP1/Ctrl)	Uniprot code	MSU (Locus ID)	Function/domain	Localization (Uniprot)	PIZSA Score	Output	GO terms
	Q9LGK6 (OsRIP1)	LOC_Os01g06740	rRNA N-glycosylase	Cytoplasm/nucleocytoplasm	–	–	Translation (GO:0006412)
3.35	Q652S1	LOC_Os06g14740	NAD-binding domain, Sugar binding domain and a nucleotide binding domain	Cytoplasm	0.372	Unstable binding	Carbohydrate metabolic process (GO:0005975)
2.83	O65037	LOC_Os08g31228	50S ribosomal protein L27	Chloroplast	N/A	N/A	Translation (GO:0006412) Structural molecule activity (GO:0005198)
2.76	P12149	LOC_Os12g34062	30S ribosomal protein S12	Chloroplast	0.844	Possible binding	Translation (GO:0006412); structural molecule activity (GO:0005198)
2.71	A0A0P0VEB3	LOC_Os02g03890	Nuclear transport factor 2 domain and an RNA recognition motif	Cytoplasm	N/A	N/A	mRNA binding (GO:0003729); RNA binding (GO:0003723)
2.48	Q6ZBV1	LOC_Os08g10020	Photosystem II 10kDa polypeptide Psbr	Chloroplast membrane	1.864	Favorable binding	Photosynthesis (GO:0015979)
2.35	Q6K439	LOC_Os09g04790	Plastid-lipid associated protein/fibrillin domain, fibrillin-like protein 2	Chloroplast	-1.594	Unstable binding	
2.32	Q8W0D1	LOC_Os05g45660	RNA-binding protein	Cytoplasm	0.682	Unstable binding	mRNA binding (GO:0003729) RNA binding (GO:0003723)
1.78	Q2R4A1	LOC_Os11g29190	40S ribosomal protein S5 (Os11g0482000)	Cytoplasm	1.359	Favorable binding	Translation (GO:0006412); structural molecule activity (GO:0005198); RNA binding (GO:0003723)
1.69	Q2QVJ6	LOC_Os12g12580	NADP-dependent oxidoreductase	Cytoplasm	0.433	Unstable binding	
1.34	Q2QLR2	LOC_Os12g43600	Glycine-rich RNA-binding protein GRP1A	Cytoplasm	-1.005	Unstable binding	mRNA binding (GO:0003729); RNA binding (GO:0003723)
1.12	P28752	LOC_Os07g38730	Tubulin alpha-1 chain, α-tubulin	Cytoplasm	0.930	Possible binding	Structural molecule activity (GO:0005198)

N/A indicates that the predicted structures for these proteins are of poor quality and cannot be used to perform protein-protein docking.

The representative pose for each complex between OsRIP1 and the target proteins underwent rescoring utilizing the Protein Interaction Z Score Assessment (PIZSA) online server (Roy et al., 2019), an empirical scoring function that assesses the stability of protein assemblies taking into consideration amino acid pair preferences. A distance threshold of 8 Å was applied, and PIZSA scores exceeding 0.8 were deemed indicative of possible binding, with values surpassing 1.0 indicating favorable binding.

### 2.13 Statistical analysis

Each treatment comprised at least three independent biological replicates. All results were presented as mean ± standard deviation (SD) except for the result of plant health parameters that were presented as mean ± standard error of mean. Data were analyzed by the t-test and one-way analysis of variance (ANOVA) using SPSS 17.0 (SPSS Inc., Chicago, IL). Multiple testing correction was performed with the Duncan correction, considering *p < 0.05, **p < 0.01, ***p < 0.001 as significance. For the analyses of the multispectral proxies, Mock and MeJA treated plants were pairwise compared as per independent sample t-test, implementing a Bonferroni multiple-significance test correction.

## 3 Results

### 3.1 Phenotypic analysis of OsRIP1-overexpressing transgenic rice plants

**Figure 1:**
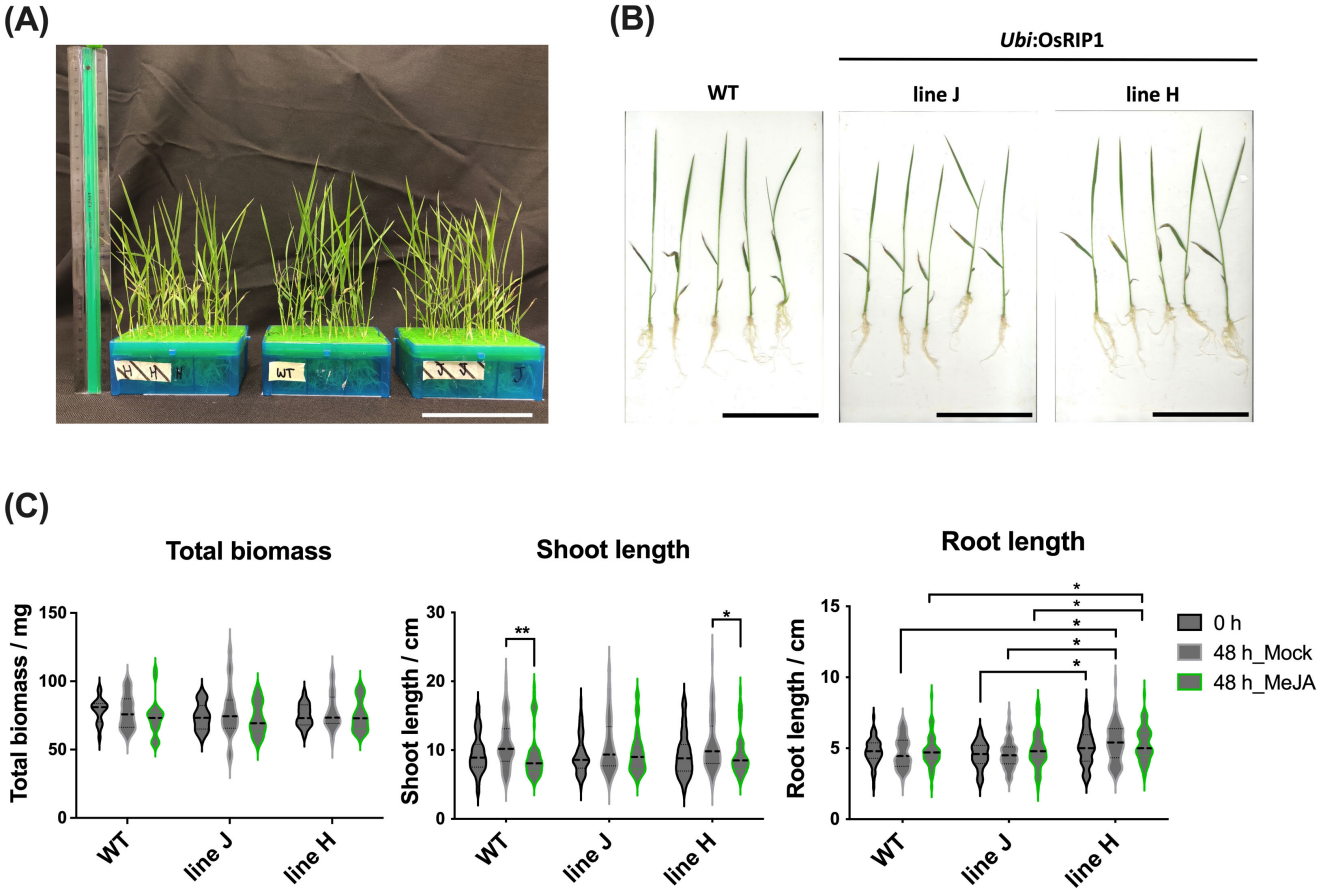
Phenotypic analysis of WT plants and T4 *OsRIP1*-OE transgenic rice plants (independent lines J and H) under normal growth conditions (0 h) and after 48 h of mock- or MeJA treatment. **(A)** Morphology of 14-day-old WT and T4 *OsRIP1*-OE rice plants hydroponically grown in the ½ Hoagland solution in 96-well tip boxes (at 0 h, before treatment). Ruler = 30 cm. **(B)** 5 individual plants of WT, lines J and H (at 0 h, before treatment). All scale bars = 10 cm. **(C)** The total biomass, shoot length and root length of plants of WT, lines J and H were measured under normal growth conditions. Each point represents means ± SD, from 60 individual plants (n = 60). The middle dotted line in the violin box refers to medians; the thin dotted lines refer to the median lower quartile and upper quartile; the width of the violin box represents the local distribution of feature values along the y axis. Statistically significant differences between points in the graphs are indicated by asterisks, *p < 0.05, **p < 0.01.

Phenotypic analysis on 14-day-old rice plants from two independent transgenic lines overexpressing OsRIP1 (*OsRIP1*-OE) revealed no visible morphological changes when compared to WT plants (**Figure 1B**). Under normal growth conditions no significant differences in total biomass and shoot length were observed for WT plants and plants from transgenic lines. Although plants from line J revealed a significantly shorter root length compared to plants from line H, no significant differences in root length were observed for line J compared to WT plants (**Figure 1C**).

MeJA exposure for 48h caused a significant inhibition of shoot growth in WT plants and plants from line H, unlike plants from line J. Moreover, plants from line H exhibited significantly longer roots compared to WT plants and line J during both normal growth conditions and MeJA exposure for 48 h (**Figure 1C**).

### 3.2 Effect of exogenously applied MeJA on health parameters of rice plants

**Figure 2:**
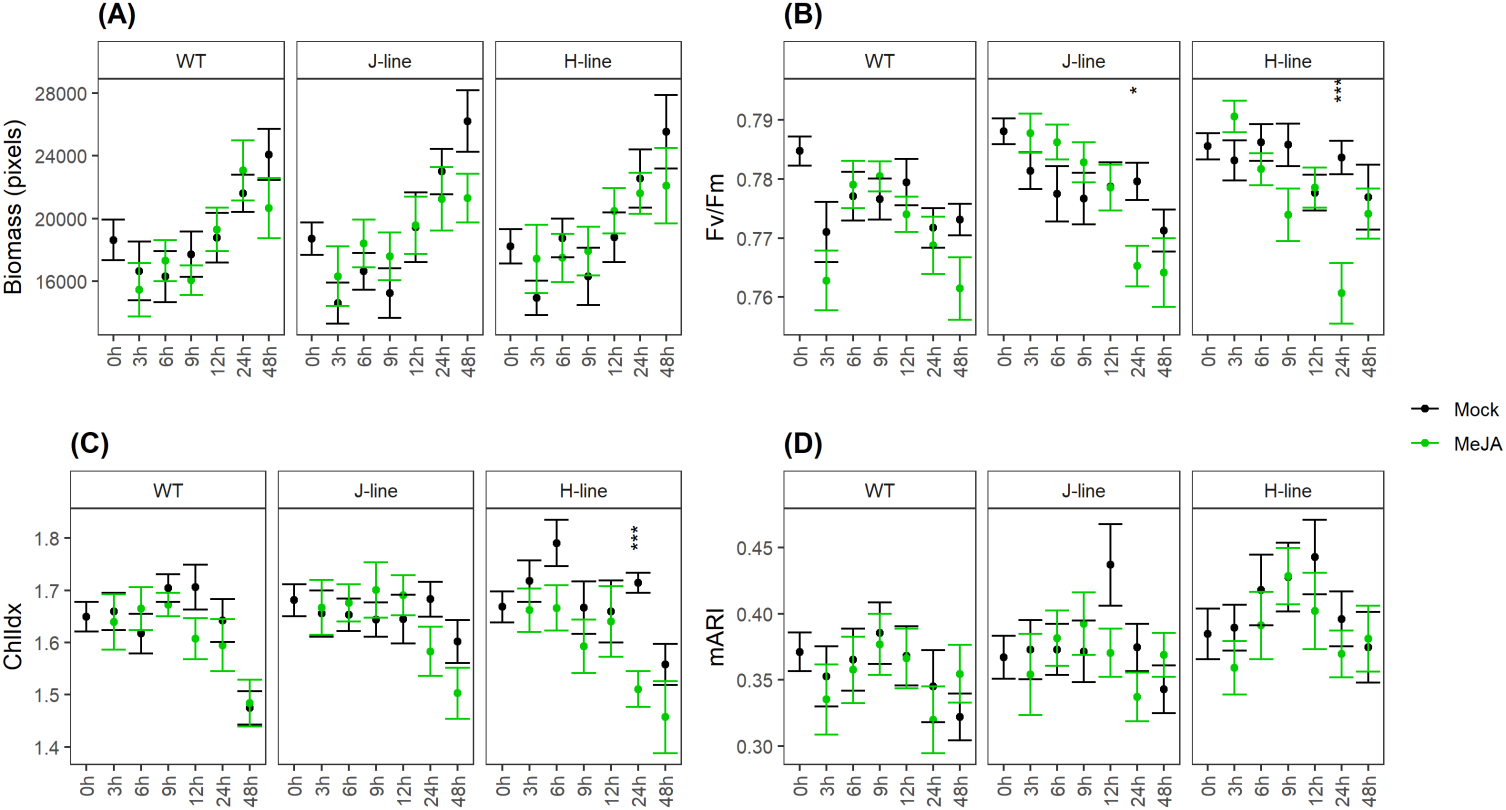
Multispectral parameters evaluated for WT plants and OsRIP1-OE plants under MeJA treatment and mock treatment based on side-view images throughout time (n = 15 plants) captured by the multispectral imaging platform. All the data represent the mean ± standard error. **(A)** estimated biomass (in pixels) of rice plants. Rice shoot biomass was evaluated as number of chlorophyll-containing pixels per plant. **(B)** chlorophyll fluorescence (Fv/Fm). **(C)** chlorophyll index (ChlIdx). **(D)** modified anthocyanin reflectance index (mARI). Significant differences (as per independent sample t-test with Bonferroni multiple-significance test-correction) between the mock and MeJA treatment are indicated by asterisks, *p < 0.05, ***p < 0.001.

To assess the effects of exogenously applied MeJA on WT plants and *OsRIP1*-OE plants of both line J and line H, the multispectral imaging platform was employed to visualize multispectral phenomics from the side view (n = 15 plants, 3 plants/image, **Supplementary Materials 1**, **Supplementary Figure S7**). All mock-treated plant groups consistently showed a non-significant increase in shoot biomass compared to the plants treated with MeJA for 48 h (**Figure 2A**; **Supplementary Materials 1**, **Supplementary Figure S8A**).

The effect of MeJA on the efficiency of photosystem II was evaluated as chlorophyll fluorescence (Fv/Fm; **Figure 2B**; **Supplementary Materials 1**, **Supplementary Figure S8B**). MeJA treatment for 3 h yielded slightly lower Fv/Fm-values in WT plants, while slightly higher Fv/Fm-values were apparent in plants of both line J and line H. MeJA treatment for 24 h resulted in significantly lower Fv/Fm-values in both line J and line H, while WT plants remained unaffected. This showed to be a transient effect since no significant differences were observed between plants of both line J and line H and WT plants after 48 h of MeJA exposure.

The effect of MeJA on the leaf chlorophyll content was evaluated as the chlorophyll index (ChlIdx; **Figure 2C**; **Supplementary Materials 1**, **Supplementary Figure S8C**). After 24 h, MeJA-treated plants from both transgenic lines showed impaired ChlIdx-values compared to the untreated (mock) plants, which was not the case for the WT plants.

Plants treated with MeJA were hallmarked by slightly lower (non-significant) mean mARI-values after 3 h for all three plant groups (**Figure 2D**; **Supplementary Materials 1**, **Supplementary Figure S8D**). This was a constant trend in WT plants as well as plants from line H up to 24 h. After 48h the opposite effect was observed, MeJA treatments resulted in slightly higher mARI values compared to mock treated plants. For plants from line J, the same trend was observed, except for timepoints 6 h and 9 h.

Transcriptome analysis and GO analysis revealed that the enrichment of “S-linalool synthase activity” (in shoots) and “terpene synthase activity” (in roots) in molecular function was characteristic for *OsRIP1*-OE plants from line J (**Supplementary Materials 1**, **Supplementary Figure S9**, **Supplementary Tables S15**, **S16**). Among these genes assigned was a monoterpene linalool encoded by *Os02g0121700* with a defensive function in plants (**Supplementary Materials 1**, **Supplementary Figure S9**; **Supplementary Table S17**).

### 3.3 OsRIP1 affects photosynthesis and cell cycle regulation in shoots under MeJA stress

**Figure 3:**
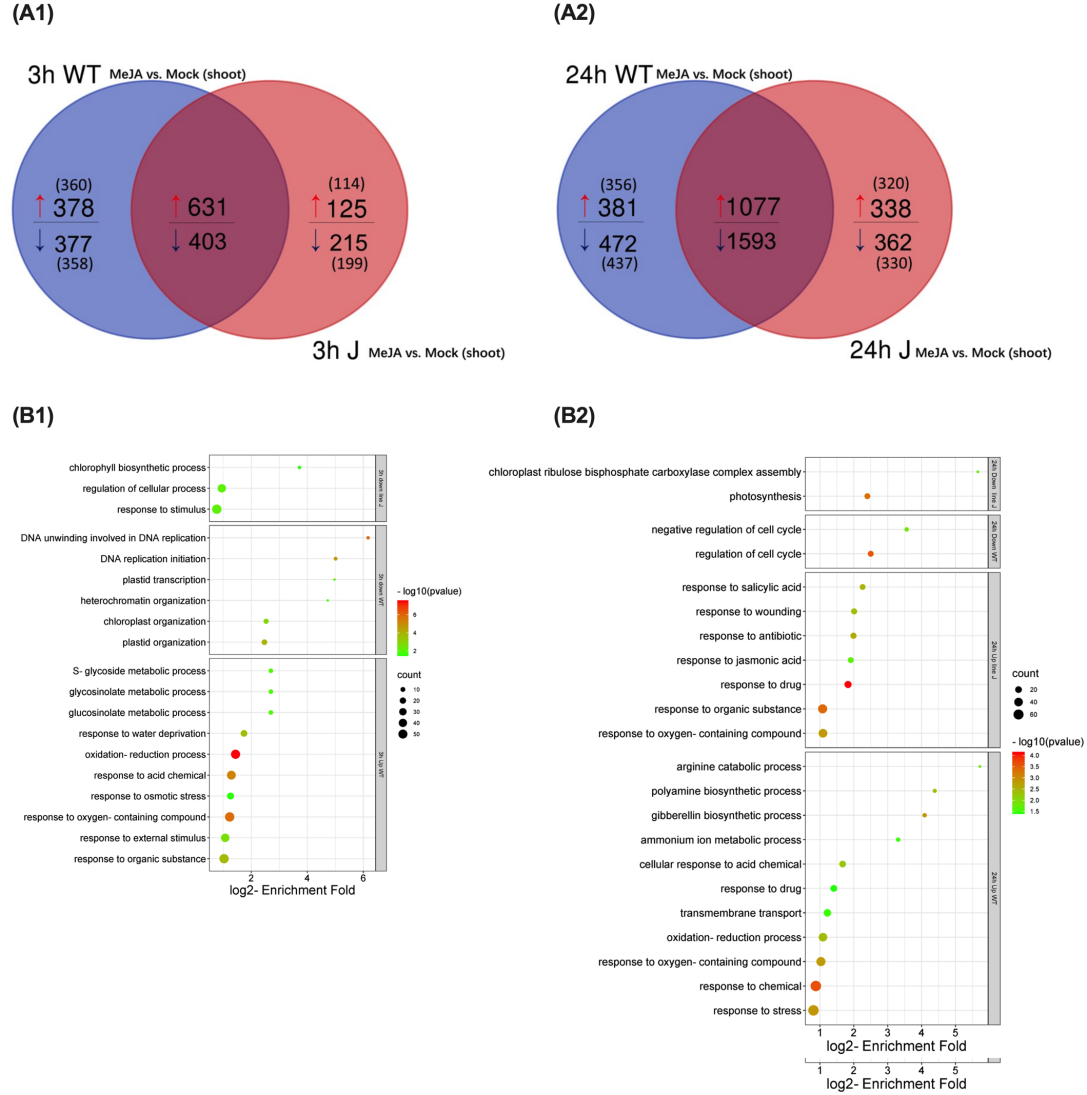
Gene ontology (GO) analysis for differentially expressed genes (DEGs) (log2 fold change [FC] > 1, log2 [FC] < -1, FDR < 0.05) unique for shoots of WT plants or *OsRIP1*-OE plants from line J after MeJA treatment (MeJA vs. mock) for 3 h and 24 h, respectively. **(A)** Venn diagram of DGEs (MeJA vs. mock) at 3 h **(A1)** or 24 h **(A2)** post MeJA treatment between WT plants and *OsRIP1*-OE plants from line J. **(B)** Enriched GO terms at 3 h **(B1)** or 24 h **(B2)** MeJA treatment in the category ‘biological process’.

mRNA sequencing was performed for shoot and root samples harvested from WT plants and T4 *OsRIP1*-OE transgenic plants from line J subjected to MeJA treatment or mock treatment. Plants from line J treated with MeJA for 3 h yielded 215 differentially expressed and down-regulated genes unique for shoots. Among these 199 annotated genes were significantly enriched in GO terms of “chlorophyll biosynthetic process”, “regulation of cellular process” and “response to stimulus” (**Figure 3B1**; **Supplementary Materials 1**, **Supplementary Table S2**). We also identified 125 differentially expressed and up-regulated genes in these shoots (**Figure 3A1**) but no Gene Ontology (GO) enrichment results were found for the 114 annotated genes.

For 358 annotated genes down-regulated only in shoots of WT plants after MeJA exposure for 3 h (MeJA vs. mock, 3 h), GO enrichment analysis indicated two biological processes, in particular “DNA unwinding involved in DNA replication” and “DNA replication initiation” with the highest p-value (**Figure 3B1**; **Supplementary Materials 1**, **Supplementary Table S3**). When rice plants were subjected to MeJA stress for 24 h, GO terms of photosynthesis-related biological processes were over-represented in 330 down-regulated annotated genes only found in shoots of plants from line J (**Figure 3A2**). GO terms related to negative regulation of cell cycle were characteristic for the set of 437 down-regulated transcripts unique in WT plants exposed to MeJA for 24 h (**Figure 3B2**; **Supplementary Table S6**). GO ontology analyses were also performed for the root samples. An enrichment of the biological processes important for N metabolism such as the cellular nitrogen compound metabolic process (GO:0034641) was found in the set of down-regulated genes in roots of WT plants after MeJA treatment for 3 h, while this phenomenon was found in roots of *OsRIP1*-OE plants from line J at 24 h after MeJA exposure (**Supplementary Materials 1**, **Supplementary Figure S6**; **Supplementary Tables S9**–**S14**).

### 3.4 Transcript analysis for JA, GA, CK, cell cycle pathways and photosynthesis in shoots

**Figure 4:**
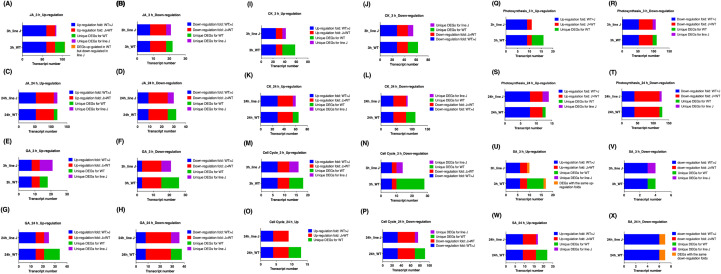
Abundance of transcripts for differentially expressed genes (DEGs) (FDR <0.05) involved in jasmonate (JA) pathway **(A–D)**, gibberellin (GA) pathway **(E–H)**, cytokinin (CK) pathway **(I–L)**, cell cycle **(M–P)**, photosynthesis **(Q–T)**, and salicylic acid (SA) pathway **(U-X)** in shoots after methyl jasmonate application for 3 or 24h. All up or down regulated DEGs were categorized into three groups, namely DEGs unique for the corresponding plants, DEGs with altered expression fold (WT > J), and DEGs with altered expression fold (J > WT). Blue and red bars represent number of transcripts identified in shoots of both WT plants and plants from line J. Bars in purple refer to number of transcripts only identified in shoots of plants from line J, while bars in green refer to number of transcripts only identified in shoots of WT plants.

Transcript numbers for genes related to JA signaling (**Figures 4A–D**), GA signaling (**Figures 4E–H**), cytokinin signaling (**Figures 4I–L**), salicylic acid signaling (**Figures 4U–X**), cell cycle (**Figures 4M–P**), and photosynthesis (**Figures 4Q–T**) were analyzed. MeJA treatment for 3 h activated many genes associated with the JA pathway. Among 82 common JA-related genes up-regulated after 3 h of MeJA treatment, 60 differentially expressed genes (DEGs) showed a higher up-regulation in WT plants compared to plants from line J (**Table 2**; **Figure 4A**). 24 up-regulated genes were unique for shoots of WT plants, while there were only 3 up-regulated unique DEGs for shoots of line J (**Table 2**; **Figure 4A**). After MeJA treatment for 24 h, a higher number of down-regulated genes were identified in shoots from line J than in WT plants (**Table 2**; **Figure 4D**), implying that OsRIP1 overexpression caused the delay of activation of the JA signaling in plants from line J compared to WT plants.

**Table 2:** Differentially expressed genes (DEGs) associated with different pathways in shoots of WT plants and plants from line J at 3 h or 24 h after MeJA treatment (MeJA-WT vs mock-WT, MeJA-line J vs. mock-line J).

Pathway	Category	Common DEGs	DEGs with regulation folds: WT>J	DEGs with regulation folds: J>WT	Unique DEGs	Total DEGs
Jasmonate signaling	3h_WT_UP*	82	60	22	24	107
	3h_line J_UP	82	60	22	3	85
	3h_WT_DOWN	18	8	10	4	22
	3h_line J_DOWN*	18	8	10	3	22
	24h_WT_UP	109	53	56	11	120
	24h_line J_UP	109	53	56	11	120
	24h_WT_DOWN	25	9	16	7	32
	24h_line J_DOWN	25	9	16	5	30
Gibberellin signaling	3h_WT_UP	13	8	5	5	18
	3h_line J_UP	13	8	5	8	21
	3h_WT_DOWN	15	3	12	11	26
	3h_line J_DOWN	15	3	12	6	21
	24h_WT_UP	21	14	7	13	34
	24h_line J_UP	21	14	7	14	35
	24h_WT_DOWN	29	8	21	9	38
	24h_line J_DOWN	29	8	21	7	36
Cytokinin signaling	3h_WT_UP	36	25	11	22	58
	3h_line J_UP	36	25	11	6	42
	3h_WT_DOWN	46	28	18	17	63
	3h_line J_DOWN	46	28	18	9	55
	24h_WT_UP	55	29	26	10	65
	24h_line J_UP	55	29	26	5	60
	24h_WT_DOWN	81	40	41	30	111
	24h_line J_DOWN	81	40	41	5	86
Cell cycle	3h_WT_UP	12	7	5	6	18
	3h_line J_UP	12	7	5	4	16
	3h_WT_DOWN	10	7	3	18	28
	3h_line J_DOWN	10	7	3	4	14
	24h_WT_UP	9	4	5	4	13
	24h_line J_UP	9	4	5	0	9
	24h_WT_DOWN	69	32	37	22	91
	24h_line J_DOWN	69	32	37	7	76
Photosynthesis	3h_WT_UP	11	9	2	5	16
	3h_line J_UP	11	9	2	0	11
	3h_WT_DOWN	97	53	44	13	110
	3h_line J_DOWN	97	53	44	10	107
	24h_WT_UP	12	8	4	1	13
	24h_line J_UP	12	8	4	2	14
	24h_WT_DOWN	120	40	80	9	129
	24h_line J_DOWN	120	40	80	7	127
Salicylic acid signaling	3h_WT_UP^**^	10	6	3	7	17
	3h_line J_UP^**^	10	6	3	0	10
	3h_WT_DOWN	3	3	0	1	4
	3h_line J_DOWN	3	3	0	1	4
	24h_WT_UP	16	9	7	6	22
	24h_line J_UP	16	9	7	1	17
	24h_WT_DOWN**	6	6	0	0	7
	24h_line J_DOWN**	6	6	0	0	7

*, one DEG up-regulated in WT but down-regulated in line J; **, one DEG with the same up-regulation or down-regulation folds in both WT plants and plants from line J.

In the set of GA-related genes at 3 h after MeJA exposure, more down-regulated genes were identified in shoots from line J than in WT plants (**Table 2**; **Figure 4F**). Compared to plants from line J, 24 h exposure to MeJA led to a higher number of up-regulated genes and less down-regulated genes associated with the GA signaling in WT plants (**Table 2**; **Figures 4G, H**), suggesting that the mRNA transcripts associated with the GA pathway occurred in lower levels in *OsRIP1* overexpressing plants than in WT plants.

Compared to plants from line J, more cytokinin-related transcripts were found to be down-regulated in WT plants after 24 h of MeJA supplementation (**Figure 4L**). A similar phenomenon was observed for genes associated with cell cycle (**Figure 4P**). It is noted that 3 h of MeJA exposure caused down-regulation of cell cycle-related genes; the number of DEGs in WT plants was double compared to plants of line J (**Figure 4N**). Among photosynthesis-related DEGs suppressed by 24 h of MeJA treatment, two-thirds of the common down-regulated genes were found to be down-regulated at a higher level in WT plants than in plants of line J (**Figure 4T**). Despite the higher number of upregulated transcripts compared to those downregulated in either line within the salicylic acid (SA) mediated signaling pathway, the total number of transcripts with differential expression was limited (< 30) (**Figures 4U–X**), and most of the SA-mediated transcripts exhibited expression changes with a log2 fold change of less than 1 (data not shown). Additionally, slight differences were observed between the MeJA-induced expression changes in SA-related genes in WT plants and those from line J. Despite the well-established crosstalk between salicylic acid and jasmonate (Peng et al., 2021), in this case, MeJA exposure did not primarily affect the SA mediated signaling pathway in either WT plants or *OsRIP1*-overexpressing plants from line J.

### 3.5 RT-qPCR validation

**Figure 5:**
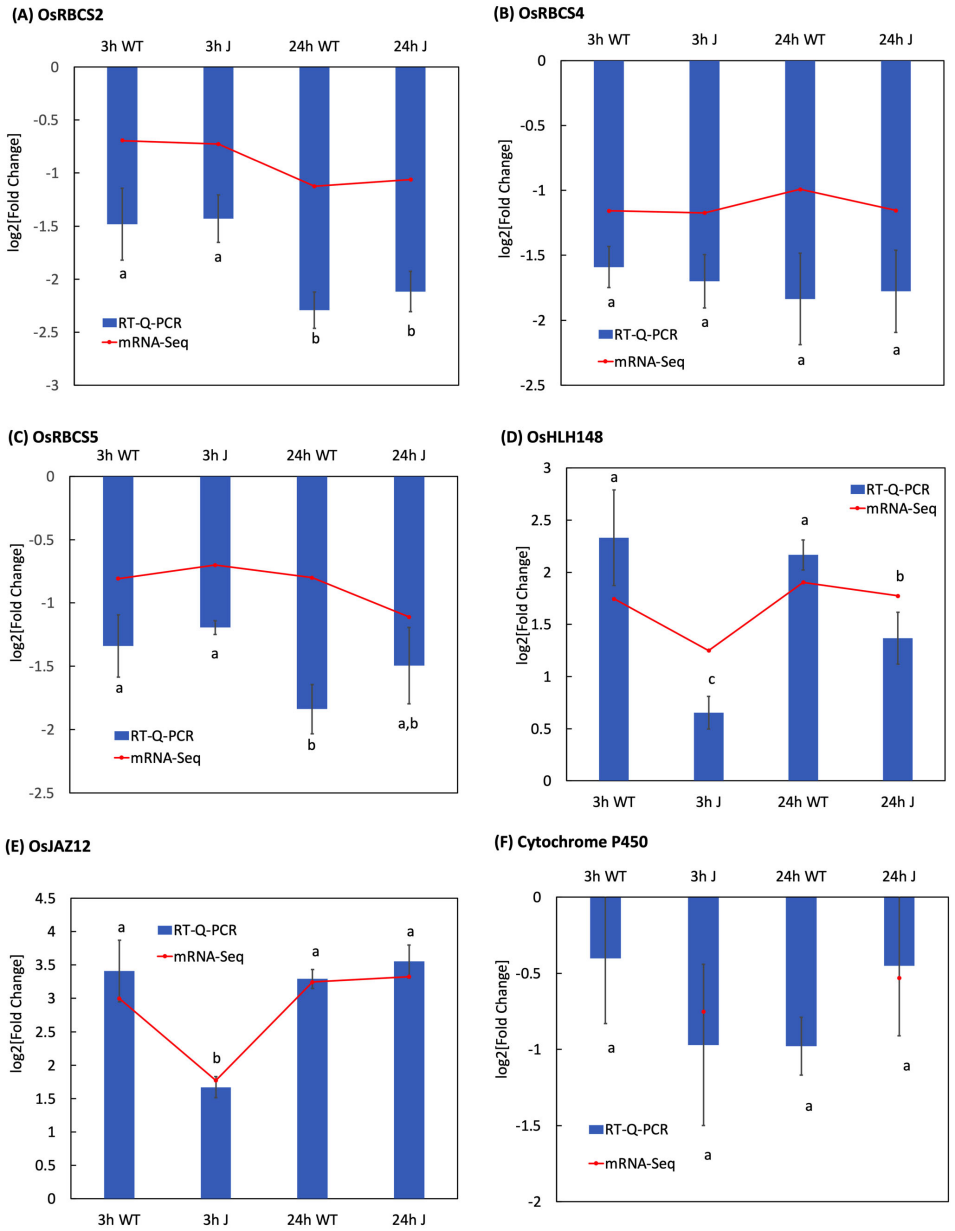
Transcript levels of genes of interest in MeJa-treated plants **(A–F)** measured using RT-qPCR relative to that in mock-treated groups (MeJa vs. mock). Each sample included three biological repeats. Three repeated biological experiments were carried out for each gene, and the error bar represents the SD of the means (n = 3). Statistically significant differences (p < 0.05) between points in the graphs are indicated with different letters; the same letters indicating no significant differences, with a>b>c.

To validate the mRNA-Seq data, 6 genes including 3 genes for photosynthesis (*OsRBCS2*, *OsRBCS4*, *OsRBCS5*), 2 genes involved in the JA signaling pathway (*OsHLH148*, *OsJAZ12*) and 1 gene encoding cytochrome P450 (*Cytochrome P450*) were selected for RT-qPCR analysis (**Supplementary Materials 1**, **Supplementary Table S1**; **Figure 5**). At 3 h after MeJA treatment, WT plants exhibited 3 more times of OsHLH148 transcript expression than plants from line J (**Figure 5D**). *OsbHLH148* transcript levels in shoots of plants from line J were more upregulated after 24 h of MeJA treatment compared to 3 h of MeJA treatment, but still significantly lower than that in WT plants (**Figure 5D**). Similarly, MeJA exposure resulted in less up-regulation of the expression of *OsJAZ12* in shoots of plants from line J compared to WT plants at 3 h (**Figure 5E**). 24 h of MeJA treatment stimulated more expression of *OsJAZ12* in plants from line J than 3 h of MeJA treatment (**Figure 5E**). MeJA application to WT plants for 3 h or 24 h had no discernible effect on *OsbHLH148* or *OsJAZ12* transcript levels (**Figures 5D, E**). These RT-qPCR expression profiles validate the mRNA-Seq data.

### 3.6 Identification of interaction partners of OsRIP1 using pull-down assays

Protein extracts from shoots of WT plants subjected to MeJA treatment were used as prey in pull-down assays to identify potential interaction partners of OsRIP1. Compared to the control group of proteins from Ni-NTA beads only treated with the plant protein extracts (**Supplementary Figure S10A**, lane 2), several proteins with a molecular weight between 17 kDa and 26 kDa appeared in the proteins from the bait samples (**Supplementary Figure S10A**, lane 1), and one additional one band of less 10 kDa was clearly present.

The three control samples and three bait samples were analyzed by LC-MS/MS runs. In all six samples 44,086 peptides were identified by LC-MS/MS system and attributed to a total of 1,443 proteins, in which 950 proteins could reliably be quantified. For the statistical analysis, protein intensities in bait samples were compared to those in control samples using a t-test approach with the settings at FDR = 0.05 and |log_2_FC| = 1 (**Supplementary Materials 2**). The Log2 fold change and the statistical significance (-log(p-value)) can be seen on the volcano plot (**Supplementary Figure S10C**), and 12 proteins in total were found to be significantly enriched in the bait samples. Of these 12 proteins, OsRIP1 was abundantly present as expected due to the OsRIP1 addition to the bait samples. Other 11 enriched proteins were considered to represent potential interaction partners for OsRIP1, and their localization as well as defined functions were summarized in **Table 1**.

Among these 11 potential interactors, four interactors were known to carry nucleotide-binding/recognition domains, of which tubulin alpha-1 chain (α-tubulin), a GTP-binding protein was associated with cytoskeleton organization. Based on the information of subcellular localization in the Uniprot database, 4 out of 11 putative interactors are localized to the chloroplast, while 7 interactors are predicted to be located in the cytoplasm. Three potential interactors were ribosomal proteins, of which only one (40S ribosomal protein S5) is present in the cytoplasm and 2 others (50S ribosomal protein L27 and 30S ribosomal protein S12) reside in the chloroplast. In addition to two ribosomal proteins from the chloroplast, two more proteins containing a transit peptide were also identified plastid lipid associated protein 2 and photosystem II 10 kDa polypeptide, respectively. The transit peptide is required for their transport across the relevant membranes from their site of synthesis in the cytoplasm. Plastid lipid associated protein 2 belongs to the plastid-lipid associated protein/fibrillin family, while photosystem II 10 kDa polypeptide is known to participate in photosynthesis (**Table 1**).

According to the GO enrichment analysis, the biological processes over-represented in this subset of 12 proteins, including OsRIP1 and its 11 putative interaction candidates, are related to “translation” (GO:0006412) and “cellular amide metabolic process” (GO:0043603), whereas “mRNA binding” (GO:0003729), “RNA binding” (GO:0003723), “structural molecule activity” (GO:0005198) and “structural constituent of ribosome” (GO:0003735) are enriched in the category molecular function (**Table 1**).

Protein-protein docking was applied to understand the molecular interactions between OsRIP1 and the putative interaction candidates identified by the pull-down assays (**Table 1**). Docking revealed a favorable binding of OsRIP1 with photosystem II 10 kDa polypeptide (**Supplementary Materials 1**; **Supplementary Figure S11A**), and 40S ribosomal protein S5 (**Supplementary Figure S11B**). Docking also suggested a binding with α-tubulin (**Supplementary Materials 1**; **Supplementary Figure S11C**), but unstable binding was predicted for the remaining proteins tested.

## 4 Discussion

Based on the previous findings showing that rice plants overexpressing *OsRIP1* show altered MeJA sensitivity the hypothesis was put forward that OsRIP1 participates in modulating the JA signaling pathway (Wytynck et al., 2021). In this study, we report that overexpression of *OsRIP1* exhibited no visible adverse effects on the normal growth of 14-day-old rice plants compared to WT plants prior to MeJA exposure (**Figure 1**). However, the reduction in shoot length was more significant in WT plants when compared to plants from line H after exogenous application of 100 μM MeJA for 48 h, but this significant growth reduction was clearly absent in plants from line J (**Figure 1C**), in agreement with previous work showing that *OsRIP1* overexpression alleviated MeJA induction of shoot inhibition (Wytynck et al., 2021). All these findings suggested that constitutive expression of *OsRIP1* plays a role in enhanced tolerance to MeJA stress.

A monoterpene linalool encoded by *Os02g0121700* was identified as characteristic for *OsRIP1*-OE plants from line J (**Supplementary Materials 1**; **Supplementary Figure S9**, **Supplementary Tables S15**–**S17**). It exerts a defensive function in plants and could boost JA biosynthesis or signaling, and up-regulate the expression of defense-related genes in rice (Taniguchi et al., 2014), tomato (López-Gresa et al., 2017), as well as *Satsuma mandarin* (Shimada et al., 2017). Therefore, the presence of “S-linalool synthase activity” in the GO molecular function category for plants from line J (**Supplementary Materials 1**; **Supplementary Figure S9**; **Supplementary Table S16**) might highlight the increase in linalool concentrations as signaling molecules to enhance and coordinate the plant defense responses in *OsRIP1*-OE plants from line J.

Even though no significant MeJA-induced suppression of the shoot biomass was observed by the multispectral imaging platform during the time-course experiment (**Figure 2A**), the leaf growth inhibition in WT plants after the addition of exogenous MeJA (**Figure 1**) may be a consequence of down-regulation in DNA replication at the early timepoint 3 h post treatment (**Figure 1B1**) and subsequent negative regulation of the cell cycle after 24 h exposure to MeJA (**Figure 1B2**). These findings were supported by substantial reports highlighting that MeJA functions as a repressor in growth (Havko et al., 2016; Guo et al., 2018), cell division (Zhang and Turner, 2008) and cell expansion (Fattorini et al., 2009). The switch from the mitotic cell cycle to the endoreduplication cycle is delayed in plant cells after MeJA application. MeJA-induced disruption in leaf growth through arresting cells in G1 phase prior to the S-phase transition has been considered to inhibit the mitotic cycle, most probably by activating critical regulators of endoreduplication and affecting the expression of key determinants of DNA replication (Noir et al., 2013). Strikingly, we identified two putative interaction partners correlated to the regulation of cell cycle, 40S ribosomal protein S5 (RPS5) and α-tubulin. RPS5 is capable of favorable binding to OsRIP1 and is a structural component of the 40S small ribosomal subunit, it is believed to participate in translation through binding to the eIF-3 (Weijers et al., 2001; Browning and Bailey-Serres, 2015). Changes in ribosome biogenesis may affect global protein synthesis which would inevitably affect plant growth and development. In *Arabidopsis* plants with a mutation in the RPS5 gene (AtRPS5A) most cell-division processes were delayed or disrupted in the heterozygous mutant, and development was completely arrested at an early embryonic stage in the homozygous mutant (Weijers et al., 2001). Moreover, 6 out of top 50 positively co-expressed genes of RPS5A (**Supplementary Materials 1**; **Supplementary Table S18**) identified on the mRNA-Seq platform using the Genevestigator tool are clustered in the regulation of cytokinin (**Supplementary Materials 1**; **Supplementary Table S2.19**). RPS5A and its co-expressed genes (except one not differentially expressed) were suppressed in WT plants at 24 h following MeJA treatment but not in *OsRIP1*-OE plants from line J (**Supplementary Materials 1**; **Supplementary Table S20**). Cytokinins are tightly related to leaf senescence, and the decrease in cytokinin levels during leaf senescence is well-documented (Hönig et al., 2018; Liu et al., 2020). The marked difference in the cell cycle pathway showed more down-regulated genes in shoots of WT plants compared to plants from line J at both 3 h and 24 h after MeJA treatment (**Table 2**; **Figures 4N, P**). Accordingly, the number of down-regulated genes related to the cytokinin pathway found in WT plants were higher than that in plants of line J after MeJA treatment for 24 h (**Figure 4L**; **Table 2**).

Molecular docking also predicted a favorable binding between α-tubulin and OsRIP1. α-tubulin participates in the biological processes of microtubule cytoskeleton organization and the mitotic cell cycle (Gaudet et al., 2011; Sheoran et al., 2014). Furthermore, mutant studies demonstrated the involvement of α-tubulin in plant growth modification in rice and *Arabidopsis* (Thitamadee et al., 2002; Sunohara et al., 2009). We hypothesize that OsRIP1 functions as a regulatory protein to antagonize MeJA-mediated shoot growth inhibition, probably through interacting with 40S ribosomal protein S5 and/or α-tubulin to modulate cytokinin-mediated leaf senescence and positive regulation of cell cycle.

Externally applied MeJA inhibited N metabolism in roots of rice plants. The biological processes important for N metabolism were suppressed in roots of WT plants after MeJA treatment for 3 h, while this phenomenon was observed in roots of *OsRIP1*-OE plants from line J at 24 h after MeJA addition (**Supplementary Materials 1**; **Supplementary Figure S6**). These observations imply that OsRIP1 delayed N regulation in rice plants under MeJA stress. N deficiency remarkably reduced plant N marker traits such as chlorophyll content, total N content and protein content (Gutiérrez et al., 2007). Partially in line with previous results, impaired chlorophyll index (ChlIdx) was characteristic of *OsRIP1*-OE plants from both lines after 24 h of MeJA treatment, while this was absent in WT plants (**Figure 2C**). The N deficiency also has an important impact on the C metabolism (Guo et al., 2017). If plants maintain an adequate photosynthesis rate, it would certainly favor an efficient production of reduced C and the subsequent efficient use of N (Bi et al., 2014), since C and N metabolism are closely linked and tightly regulated (Schofield et al., 2009). Plants under low N stress exhibited a higher sensitivity to oxidative damage resulting from excessive light and were characterized by a significant suppression of the photosynthetic capacity (Mittler, 2002; Diaz et al., 2006). In the current study, one alternative explanation for the remarkable decline in the photosystem II efficiency (Fv/Fm) observed in shoots of *OsRIP1*-OE plants (**Figure 2B**) may be that MeJA limited N metabolism in root tissues and consequently decreased availability of C and energy in systemic leaves at 24 h after MeJA treatment. MeJA was reported to elicit rapid changes in C and N dynamics in tomato (Gómez et al., 2010). In addition, nitrogen metabolism is a critical process since nitrogen is an important constituent of hormones, DNA, RNA, and proteins. After 24 h of MeJA treatment, GO terms associated with the “Ribosome” pathway were assigned for the down-regulated DEGs unique in roots of plants from line J (**Supplementary Materials 1**; **Supplementary Figure S6B3**). Oh et al. (2017) reported that protein degradation and protein translation pathways underwent extensive alterations in *Magnaporthe oryzae* under nitrogen starvation by comparative proteomic analysis. Yu et al. (2017) also established that N deficiency reduced the expression of many genes involved in translation, indicating translation is inhibited during nitrogen starvation.

The better performance of *OsRIP1*-OE plants from line J under MeJA stress might be attributed to negative regulation of photosynthesis pathways. MeJA elicitation suppressed the chlorophyll biosynthetic process at 3 h, and inhibited photosynthesis at 24 h after MeJA treatment in *OsRIP1-OE* plants from line J (**Figures 3B1, B2**). It has been firmly established that photosynthetic dysfunction is one of the typical senescence symptoms induced by MeJA application in barley (Kurowska et al., 2020), tomato (Ding et al., 2018), broccoli (Rahnamaie-Tajadod et al., 2017), and rice (Li et al., 2014). In our study, OsRIP1 confers rice tolerance to MeJA stress, which was accompanied by a large decrease in photosynthesis at the 24 h timepoint. Health parameters evaluated via the multispectral imaging platform showed that MeJA treatment at 24 h resulted in a clear rapid and transient decrease in Fv/Fm in *OsRIP1*-OE plants of both line J and line H, but not in WT plants (**Figure 2A**), which partly confirms the observations at transcriptome level (**Figures 4S, T**; **Table 2**). Notably, increasing evidence indicates that the difference in the degree of photosynthetic changes in the early stages of stresses could conversely be an indicator for the resistance level (Yang and Luo, 2021). Inoculation of powdery mildew induced a larger decline in photosynthesis in resistant barley than in susceptible barley plants (Swarbrick et al., 2006). Similar observations were also documented in tobacco upon infection with *Phytophthora nicotianae* (Scharte et al., 2005), and in *Arabidopsis* plants infected with *Pseudomonas syringae* (Bonfig et al., 2006).

A photosystem II 10 kDa polypeptide, Psbr, was identified by pull-down assays. Molecular docking predicted a preferable interaction between OsRIP1 and Psbr (**Supplementary Materials 1**; **Supplementary Figure S11A**), which function is associated with the oxygen-evolving complex of photosystem II. In contrast to Psbr that is located to the chloroplast thylakoid membrane while OsRIP1 was demonstrated to be present in the cytoplasm and the nucleus (De Zaeytijd et al., 2019). It is worth mentioning that most chloroplast proteins are synthesized in the cytoplasm and transported into the chloroplast through an orchestrated transport system (Schleiff and Becker, 2011). Psbr is a nuclear-encoded thylakoid lumenal protein (Liu et al., 2012), and we speculate that it can interact with OsRIP1 before entering the chloroplast. Further investigations are needed to proof the interactions.

OsRIP1 is suggested to predominately modulate redox-related responses (**Supplementary Materials 1**; **Supplementary Figure S12**; **Supplementary Table S21**) to MeJA treatment at 3 h by the analysis of gene regulatory network (Spurney et al., 2020) using DEGs with log_2_FC > 2. Furthermore, an upregulation of antioxidant enzymes was observed at 24 h in plants from line J (**Supplementary Materials 1**; **Supplementary Table S22**). Activation of antioxidant enzymes positively regulates plant defense systems, and a recent report showed that H_2_O_2_ application resulted in reduced oxidative stress by increasing the activity of antioxidant enzymes to protect rice plants form arsenic damage (Asgher et al., 2021).

Exogenous application of MeJA activated most JA-responsive genes (**Table 2**; **Figure 4A–D**), while approximately half of GA-responsive genes were up-regulated at both 3 h and 24 h (**Table 2**; **Figures 4E–H**). It is suggested that MeJA application activated the JA pathway to a larger extent than the GA pathway at 3 h and 24 h. Moreover, 3 h of MeJA treatment resulted in a higher activation of the JA signaling pathway in WT plants when compared to *OsRIP1*-OE plants from line J, while at 24 h of MeJA application a higher level of activation of the GA pathway was observed in WT plants compared to plants from line J. The interaction between different hormone signaling pathways in plants enables an optimal utilization of the available resources for either growth or defense. Numerous studies have revealed that JAs prioritize rice defense over GA-induced growth through negatively regulating GA signaling in herbivore-attacked plants (Yang et al., 2012; Li et al., 2015; MaChado et al., 2017). GA-JA antagonistic crosstalk has also been demonstrated in rice infected with *Xoo* (De Vleesschauwer et al., 2016) and *Meloidogyne graminicola* (Yimer et al., 2018). MaChado et al. (2017) proposed that JA indirectly represses shoot growth by antagonizing the GA pathway, perhaps through down regulation of photosynthesis based on the analysis of plants treated with exogenous JA and GA. The effect of MeJA treatment on WT plants in our study agrees with this hypothesis. In contrast to WT plants, OsRIP1 overexpression confers rice tolerance to MeJA stress through delaying the early activation of endogenous JA signaling cascades.

In summary, OsRIP1 is proposed to modulate redox-related responses at the 3 h timepoint, which may lead to further increasing the antioxidant enzyme activities and transient suppression of photosynthesis at 24 h post MeJA treatment. In addition, *OsRIP1* overexpression was suggested to modulate a lower level of the early activation of endogenous JA signaling pathway under exogenous MeJA treatment, resulting in a subsequent lower suppression of pathways involved in cytokinin signaling and cell cycle. Therefore, we speculated that OsRIP1 overexpression might be an important factor involved in the pathway determining rice tolerance to exogenous MeJA application.

## Data Availability

The mRNA-sequencing data used in this study have been submitted to the NCBI Sequence Read Archive (SRA) under BioProject ID PRJNA1104416. All other data mentioned in this article are available from the corresponding author upon reasonable request.
