# Supplementary material for: Overexpression of the ribosome-inactivating protein OsRIP1 modulates the jasmonate signaling pathway in rice
Source: Front Plant Sci. 2024 Aug 14;15:1385477. doi: 10.3389/fpls.2024.1385477 (PMC11349648; doi:10.3389/fpls.2024.1385477)
Supplement: Supplementary file 1 [file DataSheet1.docx]

**Overexpression of the ribosome-inactivating protein *OsRIP1* modulates the jasmonate signaling pathway in rice**

**Simin Chen^1†^, Noémie De Zutter^2^, Anikó Meijer^1†^, Koen Gistelinck^1^, Pieter Wytynck^1^, Isabel Verbeke^1^, Vinicius J.S. Osterne^1^, Subramanyam Kondeti^1†^, Tim De Meyer^3^, Kris Audenaert^2^, Els J.M. Van Damme^1^***

^1^Department of Biotechnology, Faculty of Bioscience Engineering, Ghent University, B-9000, Ghent, Belgium

^2^Laboratory of Applied Mycology and Phenomics, Department of Plants and Crops, Faculty of Bioscience Engineering, Ghent University, B-9000, Ghent, Belgium

^3^Department of Data Analysis & Mathematical Modelling, Ghent University, B-9000, Ghent, Belgium

*** Correspondence:**

Els J.M. Van Damme

elsjm.vandamme@ugent.be

**Supplementary information**

**Figure S1.** OsRIP1 expression in rice plants overexpressing *OsBAG4* compared to that in WT plants in the background of Japonica rice (*Oryza sativa* L) variety Zhonghua 11 (Genevestigator).

**Figure S2.** OsRIP1 expression in a mutant ebr1 compared to that in WT plants in the background of Japonica rice (*Oryza sativa* L) variety Zhonghua 11 (Genevestigator).

**Figure S3.** OsRIP1 expression in shoots of drought-treated transgenic rice plants overexpressing *OsJAZ1* (*OsJAZ1*-OE) compared to mock-treated transgenic *OsJAZ1*-OE rice plants in the background of Japonica rice (*Oryza sativa* L) variety Zhonghua 11 (Genevestigator).

**Figure S4.** OsRIP1 expression in rice plants from the Xanthomonas resistance variety cea62 compared to that in WT plants in the background of Japonica (*Oryza sativa* ssp. japonica) variety Nipponbare (Genevestigator).

**Figure S5.** *OsJAZ* (LOC_Os10g25290) expression in rice plants from the *Xanthomonas* resistance variety cea62 compared to that in WT plants in the background of Japonica (*Oryza sativa* ssp. japonica) variety Nipponbare (Genevestigator).

**Figure S6.** Gene ontology (GO) analysis in biological processes of sets of differentially expressed genes (DEGs) (log2 fold change [FC] > 1, log2 [FC] < -1, FDR < 0.05) unique in roots of WT plants or in roots of *OsRIP1*-OE plants from line J after MeJA treatment (MeJA vs. Mock) at 3 h and 24 h, respectively. **(A)** Venn diagram of DGEs at 3 h **(A1)** or 24 h **(A2)** post treatment of “MeJA vs. Mock” between WT plants and *OsRIP1*-OE plants from line J. **(B)** enriched GO terms at 3 h **(B1)** or 24 h **(B2)** for up-regulated gene lists, **(B3)** for down-regulated gene lists in the biological process category.

**Figure S7.** Representative side-view images using PathoViewer for the evaluation of health parameters of rice plants. During the process of rice cultivation for the same genotype, some plants from grew vigorously while others grew smaller. A single image containing rice plants with different growth patterns is used to represent the overall situation for plants grown under identical conditions.

**Figure S8.** Multispectral parameters evaluated on WT plants and *OsRIP1*-OE plants under MeJA treatment and mock treatment based on side-view images throughout time (n = 15 plants) captured by the multispectral imaging platform, including **(A)** estimated biomass (in pixels) of rice plants; **(B)** efficiency of photosystem II (Fv/Fm); **(C)** chlorophyll index (ChlIdx); **(D)** modified anthocyanin reflectance index (mARI).

**Figure S9.** Analysis of differentially expressed genes (DEGs) (log_2_ fold change [FC] > 1, log_2_ [FC] < -1, FDR < 0.05, line J vs. WT) in roots (A) and shoots (B) under mock treatment via mRNA-sequencing. Venn diagram of DEGs in the mock-treated groups from line J vs. WT between 3 h and 24 h, **(A)** root, **(B)** shoot. **(C)** Geno ontology (GO) analysis for common up-regulated DEGs in roots (35 annotated genes) and shoots (35 annotated genes) at 3 h and 24 h, respectively. Note that no GO terms were found in the set of common down-regulated DEGs either in roots or in shoots. BP, biological process; MF, molecular function; Count, gene number. Numbers in brackets refer to the number of genes with annotation on the platform of Monocots PLAZA 4.5. DEGs involved in each GO term are listed next to bubbles, and their descriptions are shown in Table 2.

**Figure S10. Pull-down assays for putative interaction partners of OsRIP1.** Silver staining analysis **(A)** and Western blot analysis **(B)** on 15% SDS-PAGE. The protein patterns of samples were visualized in the following order, lane 1: bait sample after pull-down assays; lane 2: control sample after pull-down assays; lane 3: unbound protein fraction from bait sample in lane 1; lane 4: unbound protein fraction from control sample in lane 2; lane 5: wash fraction containing 50 mM imidazole from bait sample in lane 1; lane 6: wash fraction containing 50 mM imidazole from control sample in lane 2; lane 7: 16.62 μg of plant protein extracts; lane 8: 1 μg of purified recombinant OsRIP1 (31.4 kDa). Western blot analysis was performed using an anti-HIS antibody (1:1000, Thermo Fisher Scientific). PageRuler™ Prestained Protein Ladder (1:10,000, Thermo Fisher Scientific) was loaded as a reference. For the lanes with proteins released from beads, 12.5 μl from a total of 25 μl harvested beads were loaded. For the wash and unbound protein fractions 15 μl of samples were loaded, representing 10% of the liquid from one wash step or 1.5% of the unbound protein fraction. The red asterisk refers to the OsRIP1 polypeptide, while red arrows indicate potential interaction partners. **(C)** Volcano plot of proteins identified by LC/MS-MS (N = 3). Recombinant OsRIP1 with the His-tag is marked in blue, 11 significantly enriched proteins in OsRIP1-treated samples are indicated in green, those significantly enriched proteins in control samples are indicated in red, and non-significantly enriched proteins are shown in grey.

**Figure S11.** Modeling of protein binding between OsRIP1 and its putative interaction partners identified by pull-down assays. **(A)** Photosystem II 10 kDa polypeptide. **(B)** 40S ribosomal protein S5. **(C)** Tubulin alpha-1 chain.

**Figure S12.** Analysis of the gene regulatory network for OsRIP1 at 3 h using differentially expressed genes with log_2_FC > 2. CYP76M2 (Os08g0508000), cytokinin-O-glucosyltransferase 2 (Os02g0755900) and ICL (Os070529000).

**Table S1.** Primers of genes of interest for RT-qPCR analysis

**Table S2.** GO analysis of 199 annotated out of 215 down-regulated differentially expressed genes (DEGs) (log2FC<-1) unique in shoots of plants from line J at 3 h (MeJA-line J vs. Mock-line J)

**Table S3.** GO analysis of 358 annotated out of 377 down-regulated differentially expressed genes (DEGs) (log_2_FC < -1) unique in shoots of WT plants at 3 h (MeJA-WT vs. Mock-WT)

**Table S4.** GO analysis of 360 annotated out of 378 up-regulated differentially expressed genes (DEGs) (log_2_FC > 1) unique in shoots of WT plants at 3 h (MeJA-WT vs. Mock-WT)

**Table S5.** GO analysis of 330 annotated out of 362 down-regulated differentially expressed genes (DEGs) (log_2_FC < -1) unique in shoots of plants from line J at 24 h (MeJA-line J vs. Mock-line J)

**Table S6.** GO analysis of 437 annotated out of 472 down-regulated differentially expressed genes (DEGs) (log_2_FC < -1) unique in shoots of WT plants at 24 h (MeJA-WT vs. Mock-WT)

**Table S7.** GO analysis of 320 annotated out of 338 up-regulated differentially expressed genes (DEGs) (log_2_FC > 1) unique in shoots of plants from line J at 24 h (MeJA-line J vs. Mock-line J)

**Table S8.** GO analysis of 356 annotated out of 381 up-regulated differentially expressed genes (DEGs) (log_2_FC > 1) unique in shoots of WT plants at 24 h (MeJA-WT vs. Mock-WT)

**Table S9.** GO analysis of 694 annotated out of 747 down-regulated differentially expressed genes (DEGs) (log_2_FC < -1) unique in roots of WT plants at 3 h (MeJA-WT vs. Mock-WT)

**Table S10.** GO analysis of 449 annotated out of 477 up-regulated differentially expressed genes (DEGs) (log2FC > 1) unique in roots of WT plants at 3 h (MeJA-WT vs. Mock-WT)

**Table S11.** GO analysis of 521 annotated out of 587 up-regulated differentially expressed genes (DEGs) (log_2_FC > 1) unique in roots of plants from line J at 24 h (MeJA-line J vs. Mock-line J)

**Table S12.** GO analysis of 437 annotated out of 472 up-regulated differentially expressed genes (DEGs) (log_2_FC > 1) unique in roots of WT plants at 24 h (MeJA-WT vs. Mock-WT)

**Table S13.** GO analysis of 641 annotated out of 691 down-regulated differentially expressed genes (DEGs) (log_2_FC < -1) unique in roots of plants from line J at 24 h (MeJA-line J vs. Mock-line J)

**Table S14.** GO analysis of 943 annotated out of 1017 down-regulated differentially expressed genes (DEGs) (log_2_FC < -1) unique in roots of WT plants at 24 h (MeJA-WT vs. Mock-WT)

**Table S15.** GO analysis of 35 common differentially expressed genes (DEGs) (log_2_FC>1) in root samples at both 3 h and 24h (Mock-line J vs. Mock-WT)

**Table S16.** GO analysis of 35 common differentially expressed genes (DEGs) (log_2_FC>1) in shoot samples at both 3 h and 24h (Mock-line J vs. Mock-WT)

**Table S17.** Differentially expressed genes (DEGs) involved in GO terms shown in Figure S9

**Table S18.** 50 positively co-expressed genes of 40S ribosomal protein S5 (RPS5A) identified by Genevestigator on the mRNA-Seq platform

**Table S19.** 6 out of top 50 positively co-expressed genes of RPS5A clustered in the biological process of response to cytokinin (GO:0009735)

**Table S20.** Expression levels of differentially expressed genes (DEGs) clustered in the biological process of response to cytokinin (GO:0009735) (MeJA vs. Mock)

**Table S21.** Genes involved in the gene regulatory network for OsRIP1 at 3 h (shown in Figure S11)

**Table S22.** Differentially expressed genes (DEGs) (log_2_FC > 2) unique in shoots of plants from line J at 24 h (MeJA-line J vs. mock-line J) clustered in the molecular function of antioxidant activity (GO:0016209)

## **Supplementary figures**


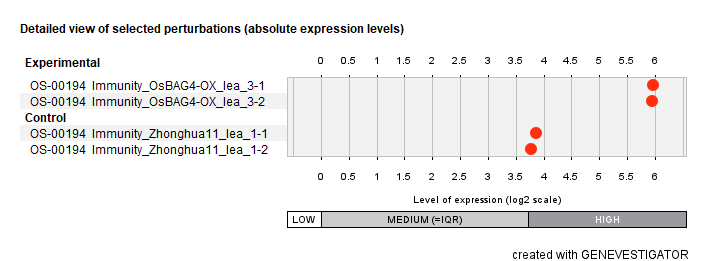


**Figure S1.** OsRIP1 expression in rice plants overexpressing OsBAG4 compared to that in WT plants in the background of Japonica rice (*Oryza sativa* L) variety Zhonghua 11 (Genevestigator).


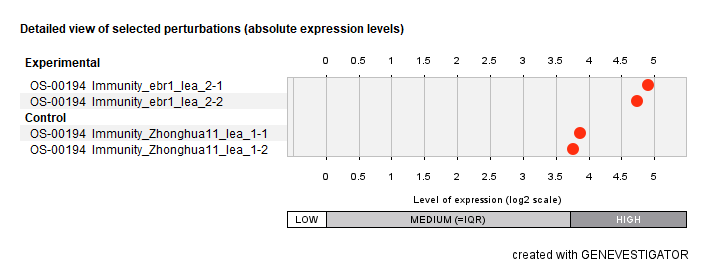


**Figure S2.** OsRIP1 expression in a mutant ebr1 compared to that in WT plants in the background of Japonica rice (*Oryza sativa* L) variety Zhonghua 11 (Genevestigator).


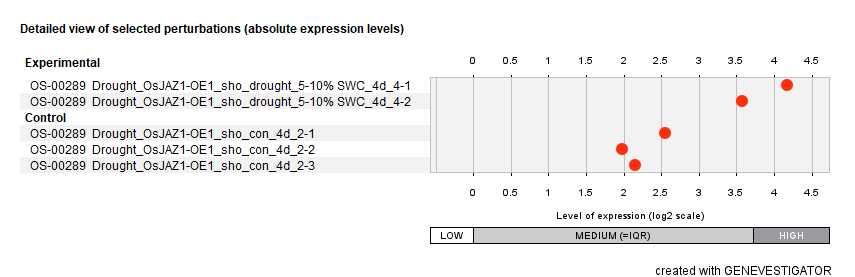


**Figure S3.** OsRIP1 expression in shoots of drought-treated transgenic rice plants overexpressing *OsJAZ1* (*OsJAZ*1-OE) compared to mock-treated transgenic *OsJAZ1*-OE rice plants in the background of Japonica rice (*Oryza sativa* L) variety Zhonghua 11 (Genevestigator).


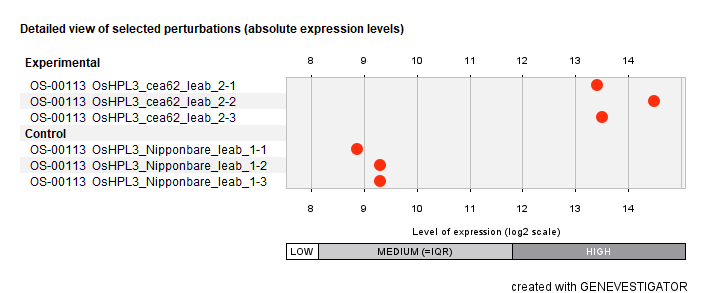


**Figure S4.** OsRIP1 expression in rice plants from the Xanthomonas resistance variety cea62 compared to that in WT plants in the background of Japonica (*Oryza sativa* ssp. japonica) variety Nipponbare (Genevestigator).


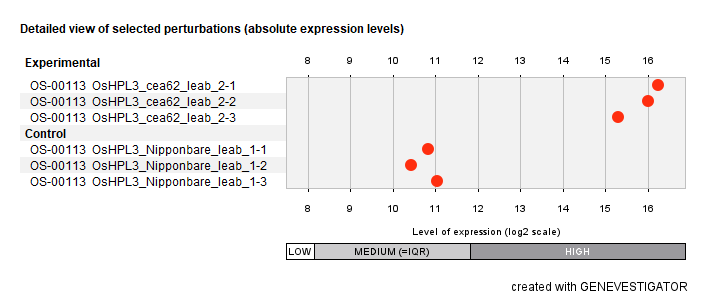


**Figure S5.** *OsJAZ* (LOC_Os10g25290) expression in rice plants from the Xanthomonas resistance variety cea62 compared to that in WT plants in the background of Japonica (*Oryza sativa* ssp. japonica) variety Nipponbare (Genevestigator).

**(A1)**


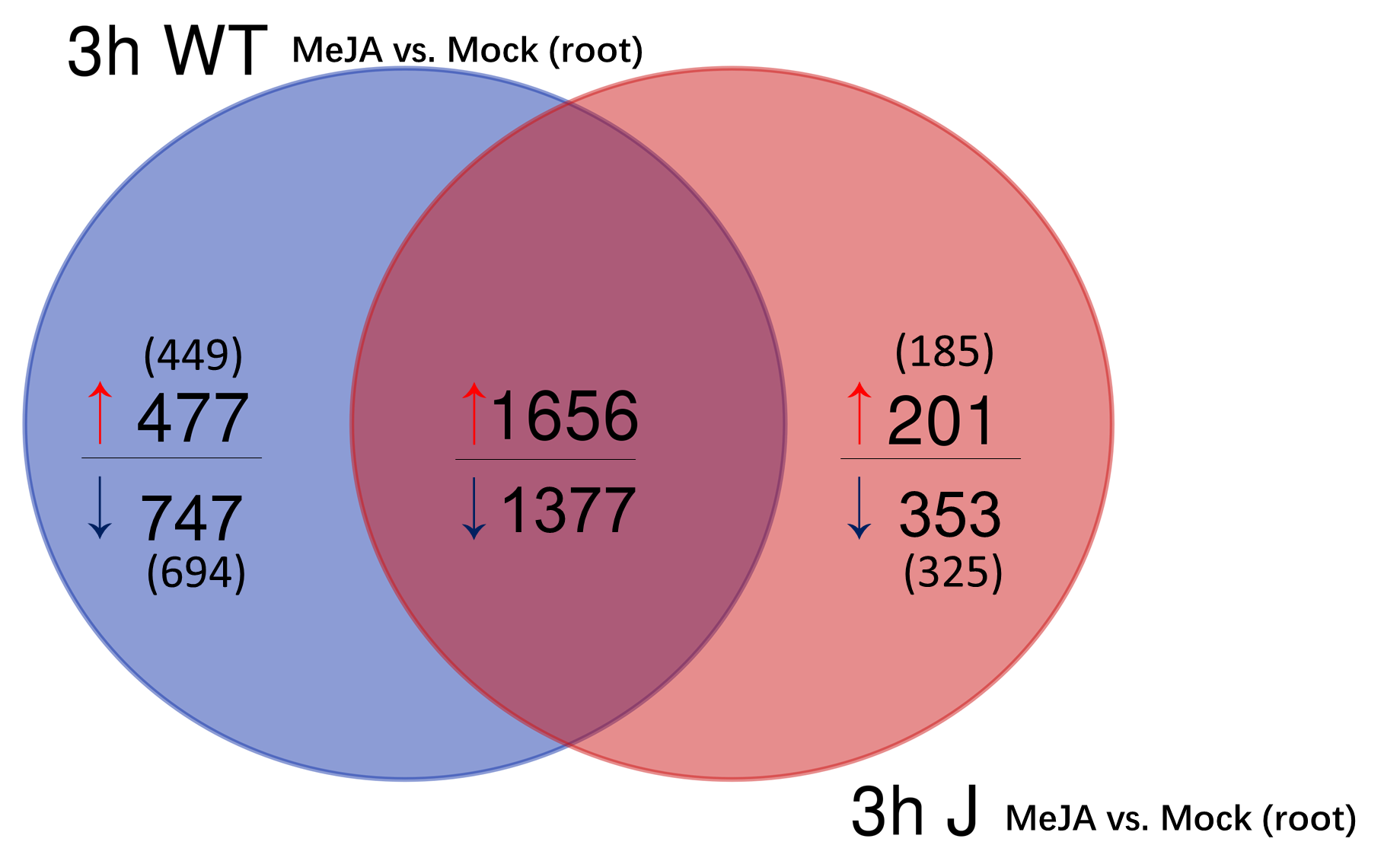


**(B1)**


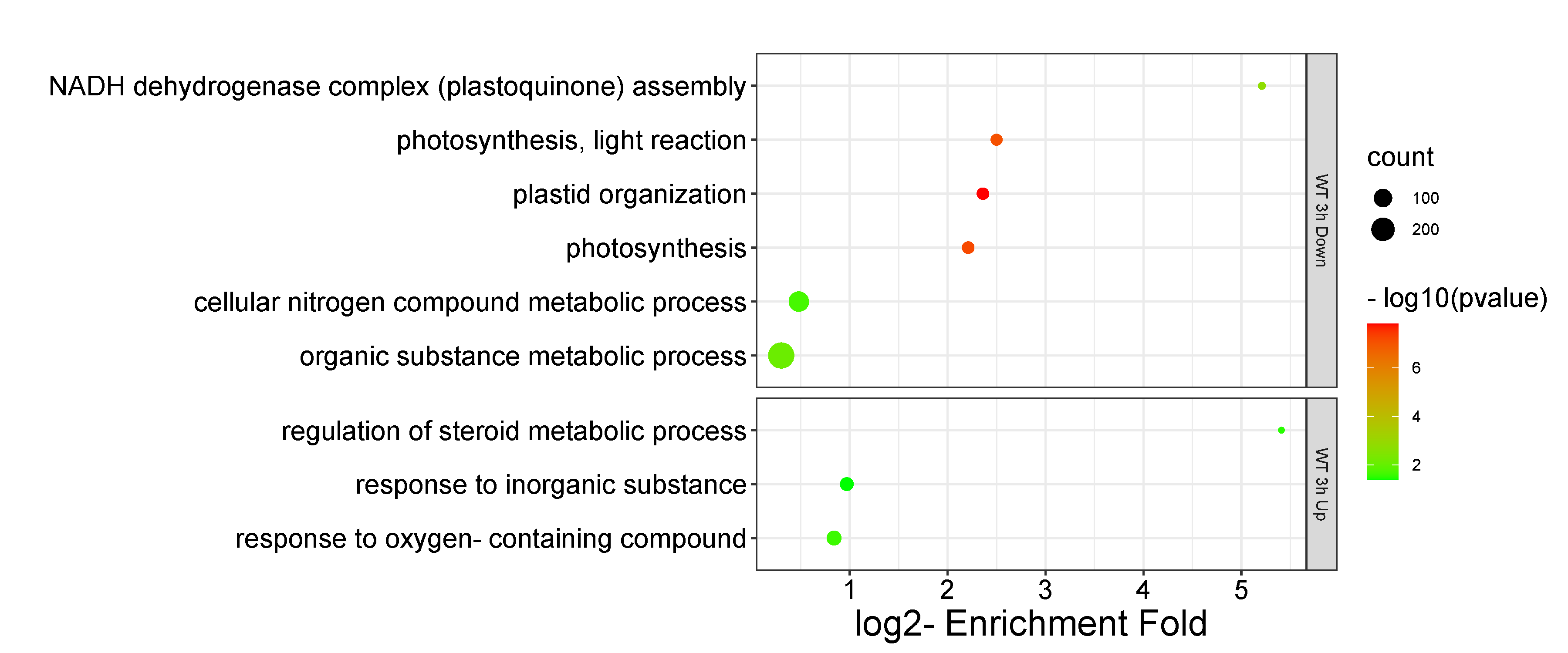


**(A2)**


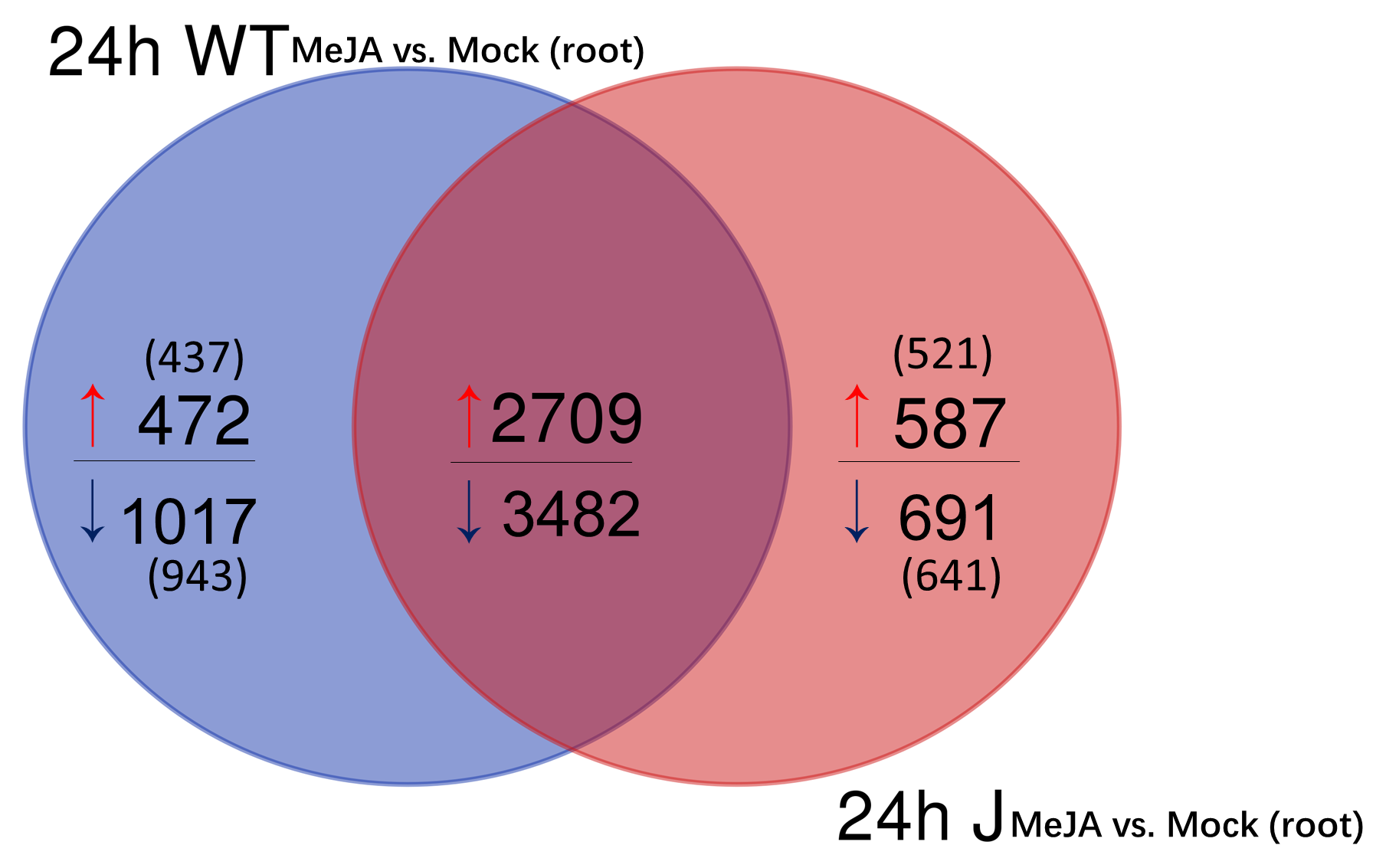


**(B2)**


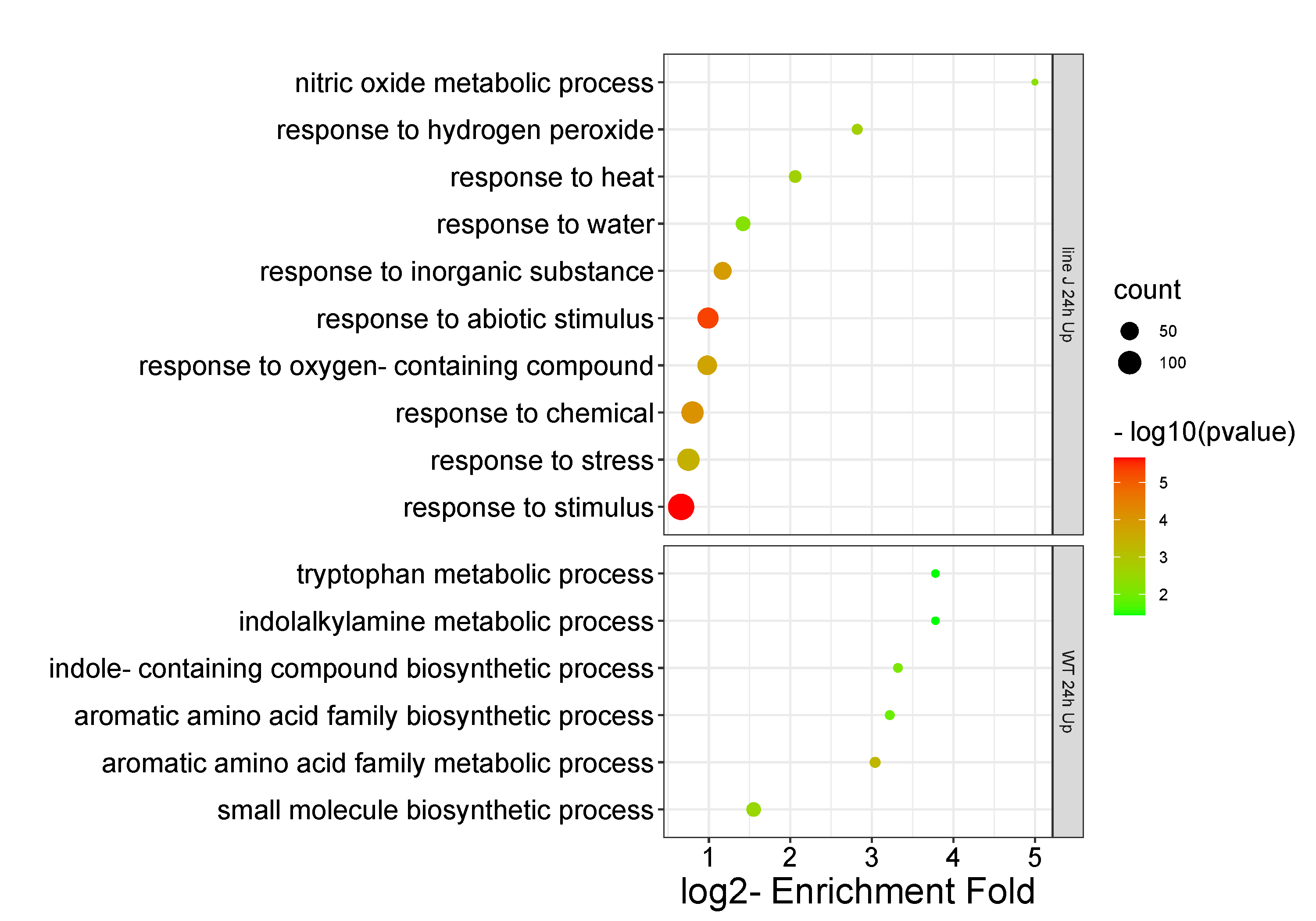


**(B3)**


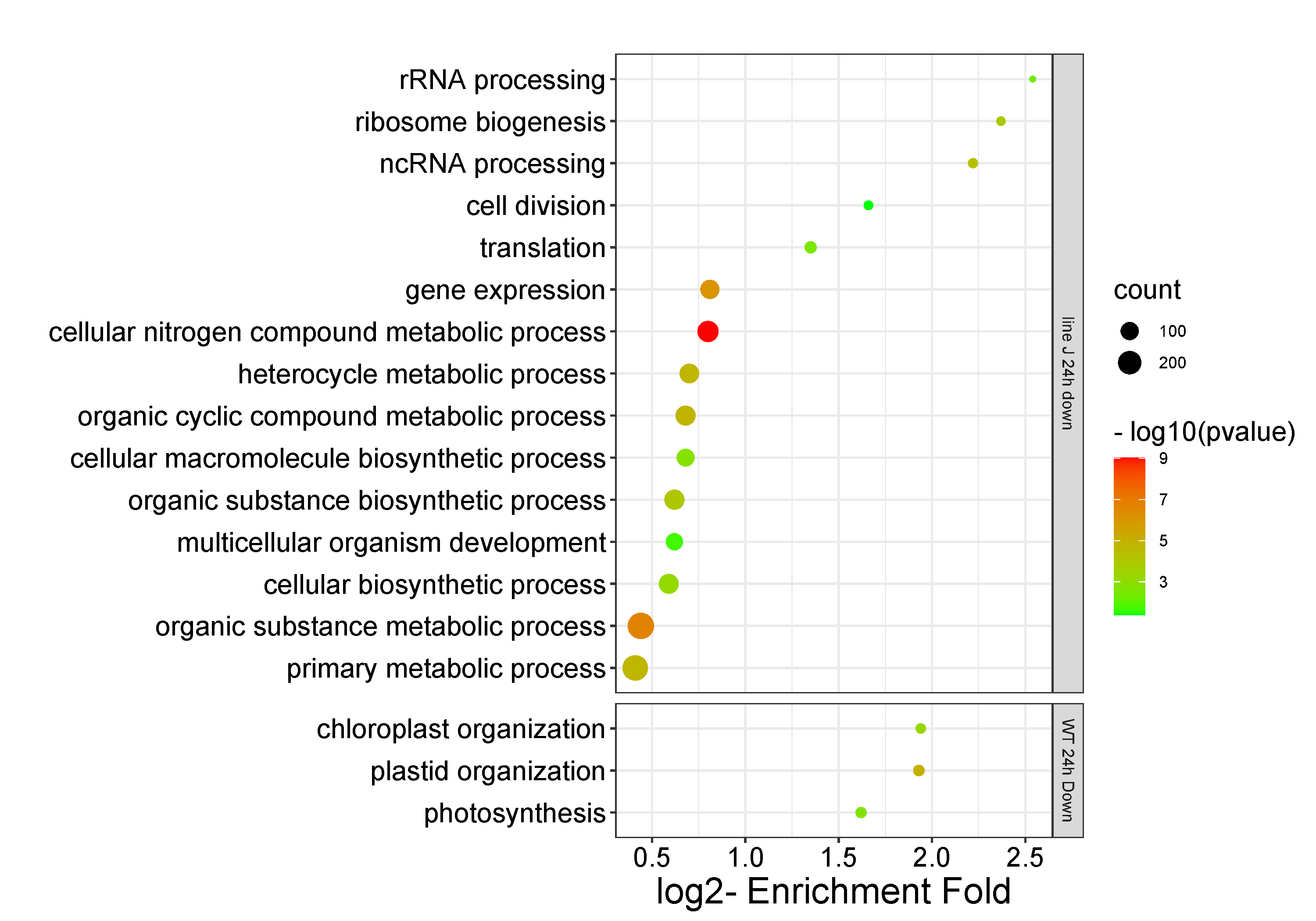


**Figure S6.** Gene ontology (GO) analysis in biological processes of sets of differentially expressed genes (DEGs) (log2 fold change [FC] > 1, log2 [FC] < -1, FDR < 0.05) unique in roots of WT plants or in roots of *OsRIP1*-OE plants from line J after MeJA treatment (MeJA vs. Mock) at 3 h and 24 h, respectively. **(A)** Venn diagram of DGEs at 3 h **(A1)** or 24 h **(A2)** post treatment of “MeJA vs. Mock” between WT plants and *OsRIP1*-OE plants from line J. **(B)** enriched GO terms at 3 h **(B1)** or 24 h **(B2)** for up-regulated gene lists, **(B3)** for down-regulated gene lists in the biological process category.


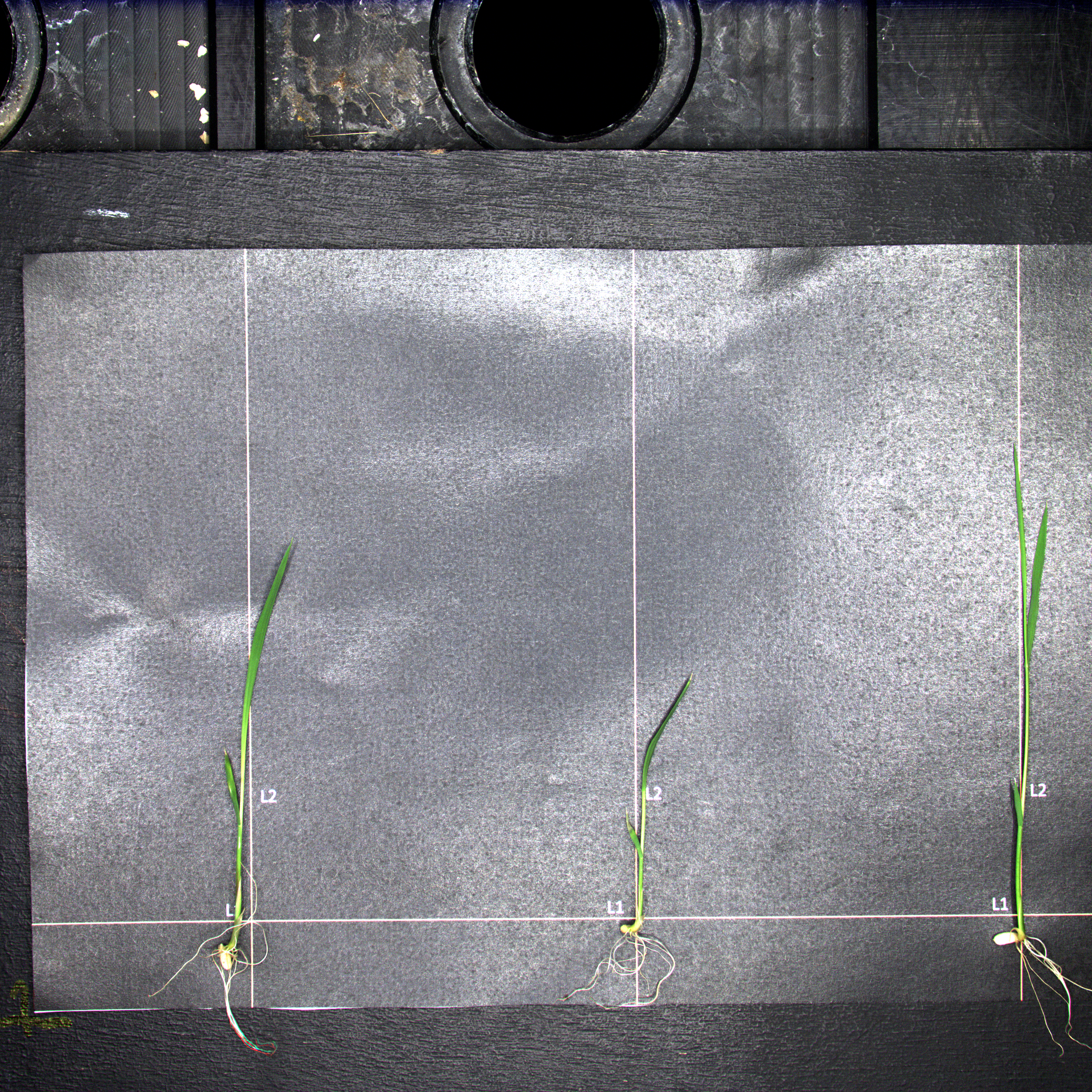


**Figure S7.** Representative side-view images using PathoViewer for the evaluation of health parameters of rice plants. During the process of rice cultivation for the same genotype, some plants from grew vigorously while others grew smaller. A single image containing rice plants with different growth patterns is used to represent the overall situation for plants grown under identical conditions.


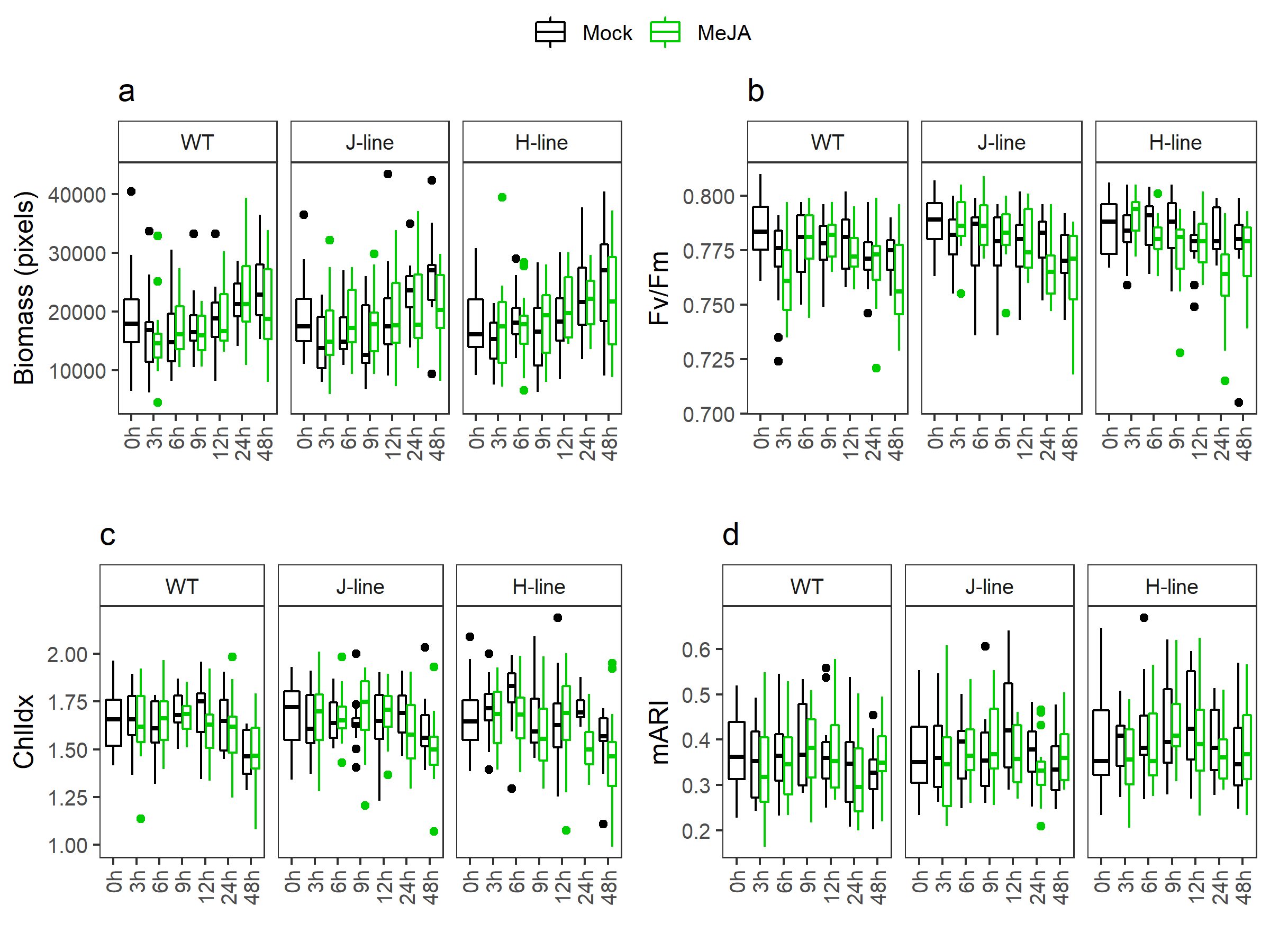


**Figure S8.** Multispectral parameters evaluated on WT plants and *OsRIP1*-OE plants under MeJA treatment and mock treatment based on side-view images throughout time (n = 15 plants) captured by the PathoViewer platform, including **(A)** estimated biomass (in pixels) of rice plants; **(B)** efficiency of photosystem II (Fv/Fm); **(C)** chlorophyll index (ChlIdx); **(D)** modified anthocyanin reflectance index (mARI).

**(A) (B)**


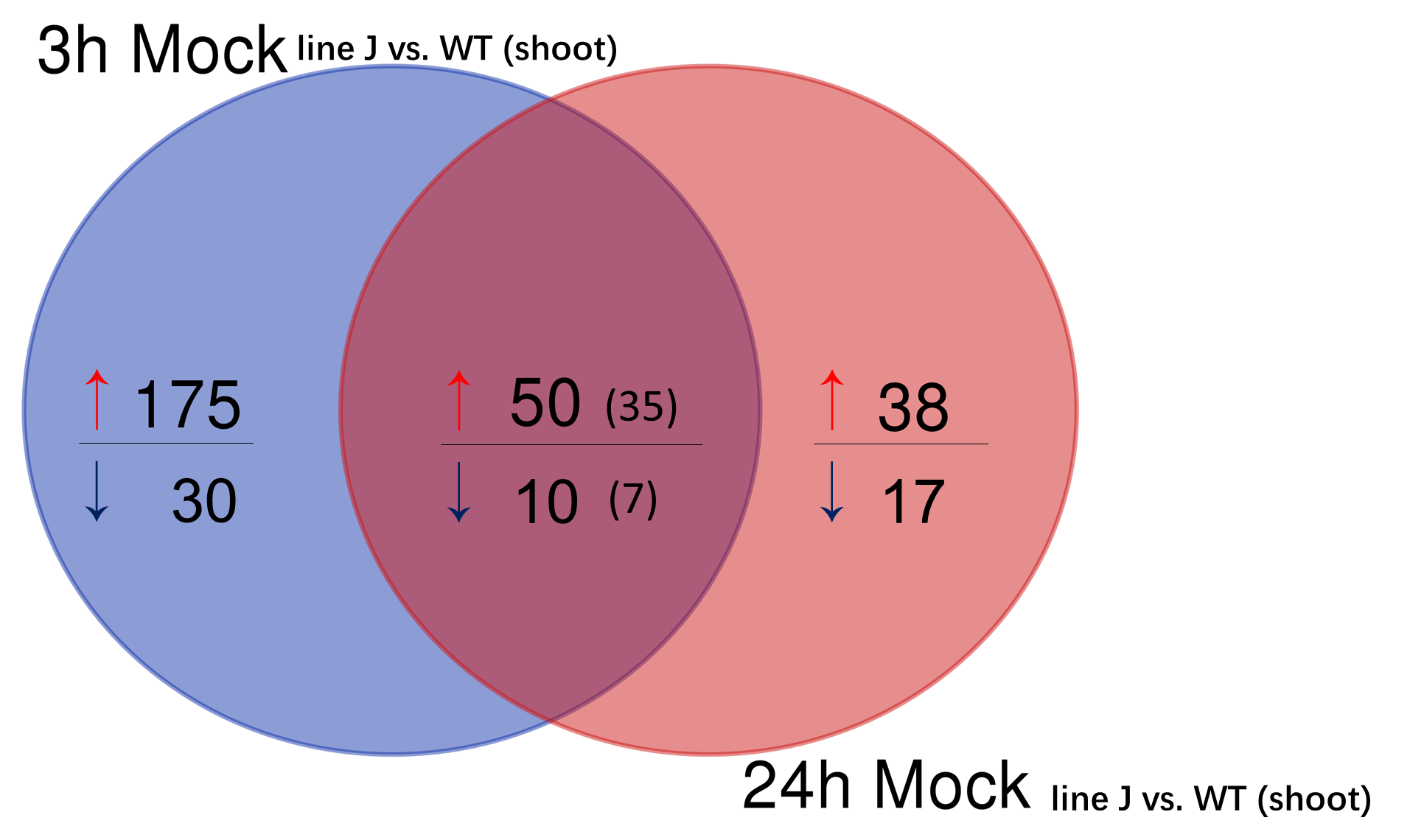

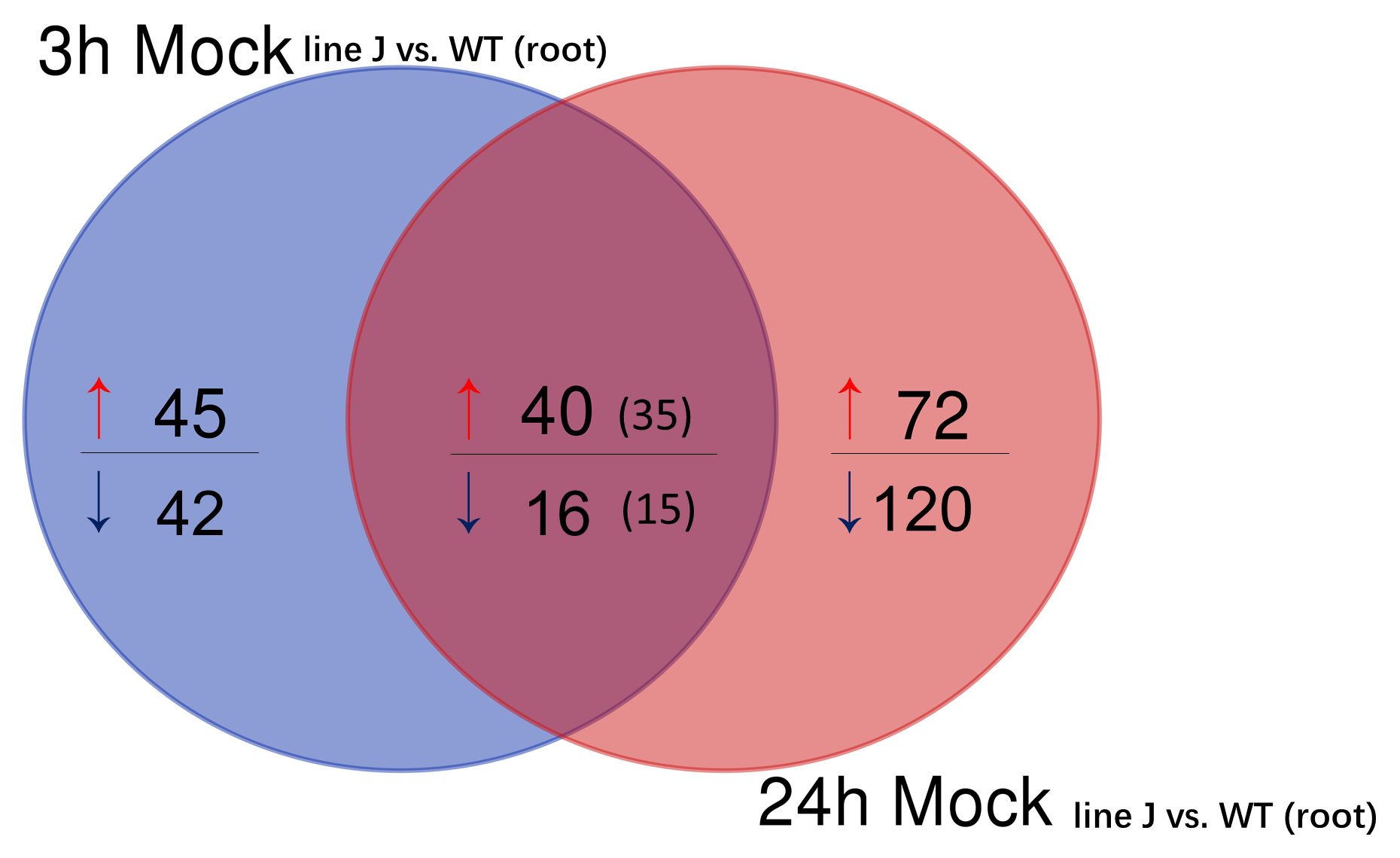


**(C)**


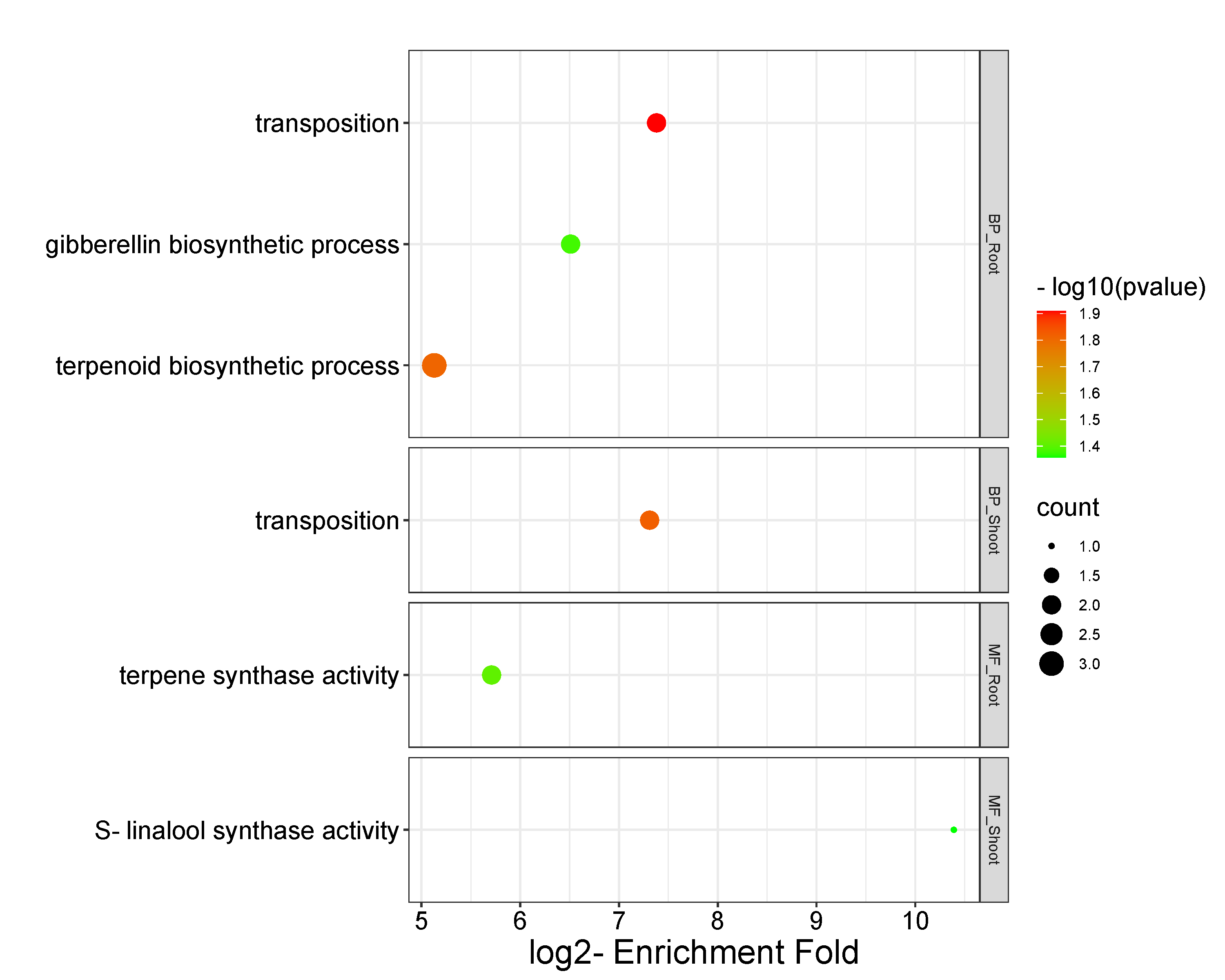


Os02g0121700

Os04g0178300

Os11g0475000

Os05g0438201

Os07g0153150

Os04g0178300

Os04g0179100

Os11g0475000

Os04g0178300

Os11g0475000

Os05g0438201

Os07g0153150

**Figure S9.** Analysis of differentially expressed genes (DEGs) (log2 fold change [FC] > 1, log2 [FC] < -1, FDR < 0.05, line J vs. WT) in roots **(A)** and shoots **(B)** under mock treatment via mRNA-sequencing. Venn diagram of DEGs in the mock-treated groups from line J vs. WT between 3 h and 24 h, **(A)** root, **(B)** shoot. **(C)** Geno ontology (GO) analysis for common up-regulated DEGs in roots (35 annotated genes) and shoots (35 annotated genes) at 3 h and 24 h, respectively. Note that no GO terms were found in the set of common down-regulated DEGs either in roots or in shoots. BP, biological process; MF, molecular function; Count, gene number. Numbers in brackets refer to the number of genes with annotation on the platform of Monocots PLAZA 4.5. DEGs involved in each GO term are listed next to bubbles, and their descriptions are shown in Table 2.


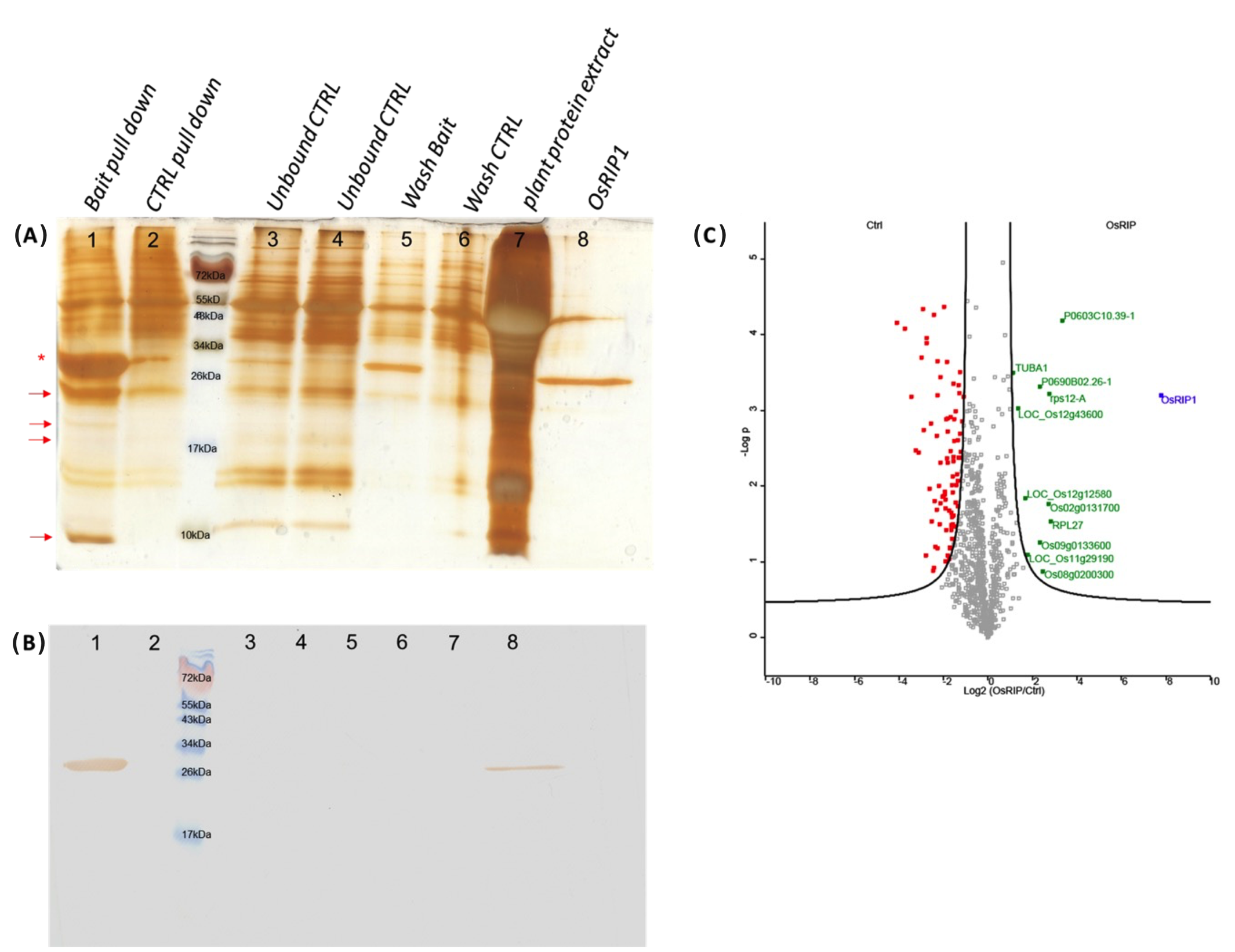


**Figure S10. Pull-down assays for putative interaction partners of OsRIP1.** Silver staining analysis **(A)** and Western blot analysis **(B)** on 15% SDS-PAGE. The protein patterns of samples were visualized in the following order, lane 1: bait sample after pull-down assays; lane 2: control sample after pull-down assays; lane 3: unbound protein fraction from bait sample in lane 1; lane 4: unbound protein fraction from control sample in lane 2; lane 5: wash fraction containing 50 mM imidazole from bait sample in lane 1; lane 6: wash fraction containing 50 mM imidazole from control sample in lane 2; lane 7: 16.62 μg of plant protein extracts; lane 8: 1 μg of purified recombinant OsRIP1 (31.4 kDa). Western blot analysis was performed using an anti-HIS antibody (1:1000, Thermo Fisher Scientific). PageRuler™ Prestained Protein Ladder (1:10,000 Thermo Fisher Scientific) was loaded as a reference. For the lanes with proteins released from beads, 12.5 μl from a total of 25 μl harvested beads were loaded. For the wash and unbound protein fractions 15 μl of samples were loaded, representing 10% of the liquid from one wash step or 1.5% of the unbound protein fraction. The red asterisk refers to the OsRIP1 polypeptide, while red arrows indicate potential interaction partners. **(C)** Volcano plot of proteins identified by LC/MS-MS (N = 3). Recombinant OsRIP1 with the His-tag is marked in blue, 11 significantly enriched proteins in OsRIP1-treated samples are indicated in green, those significantly enriched proteins in control samples are indicated in red, and non-significantly enriched proteins are shown in grey.


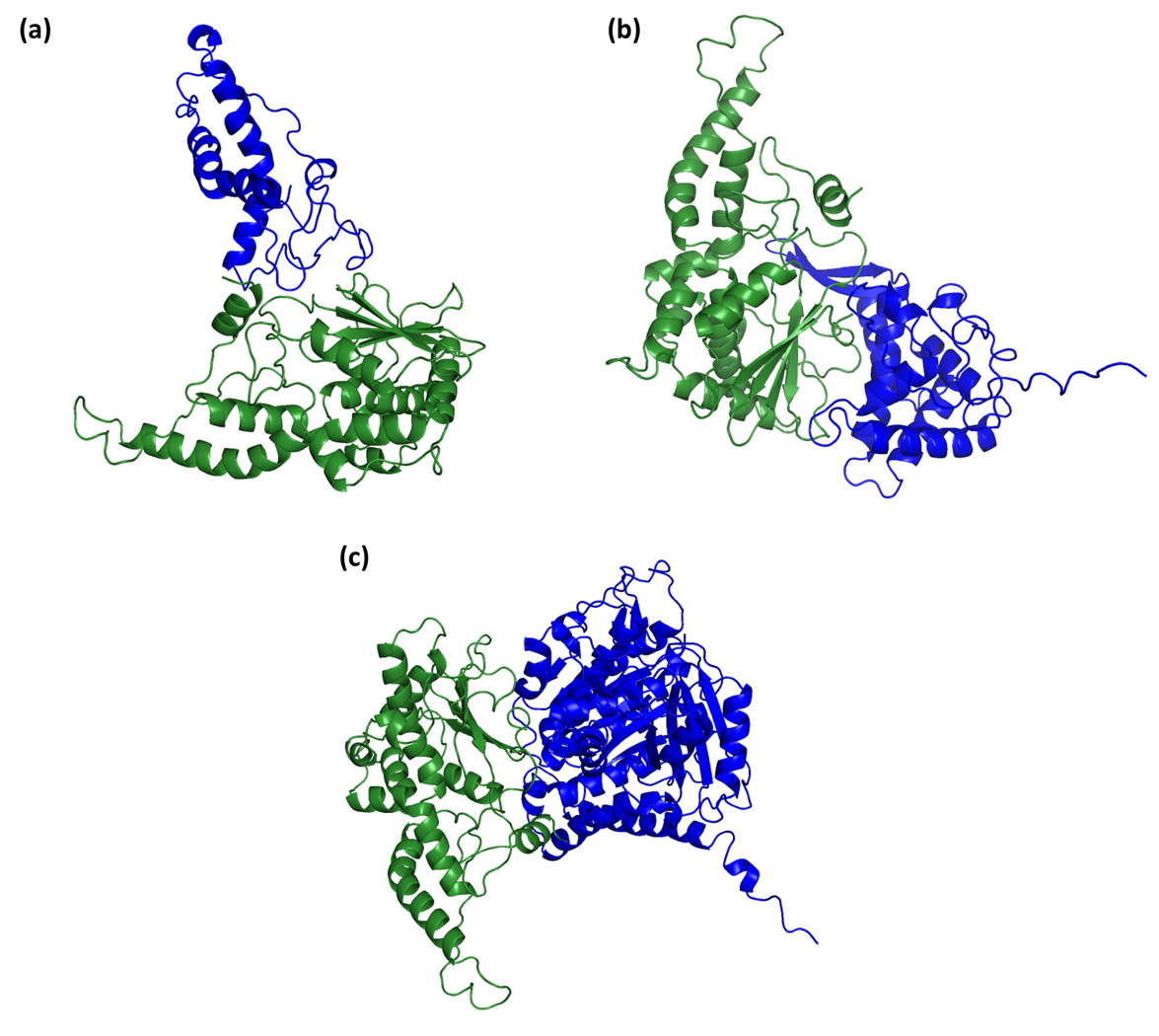


**Figure S11.** Modeling of protein binding between OsRIP1 and its putative interaction partners identified by pull-down assays. **(A)** Photosystem II 10 kDa polypeptide. **(B)** 40S ribosomal protein S5. **(C)** Tubulin alpha-1 chain.


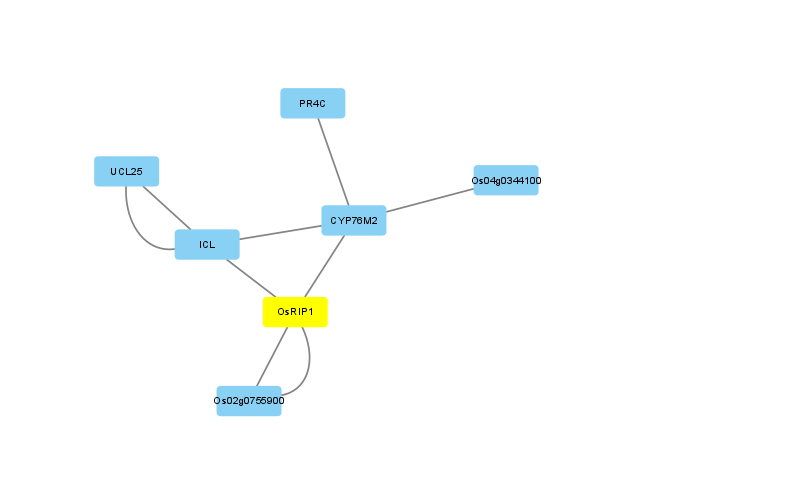


**Figure S12.** Analysis of the gene regulatory network for OsRIP1 at 3 h using differentially expressed genes with log_2_FC > 2. CYP76M2 (Os08g0508000), cytokinin-O-glucosyltransferase 2 (Os02g0755900) and ICL (Os070529000).

## **Supplementary tables**

**Table S1.** Primers of genes of interest for RT-qPCR analysis

| Category | Gene | Primer sequence | Size of amplicon | Efficiency | Correlation coefficients (R^2^) |
| --- | --- | --- | --- | --- | --- |
| **Photosynthesis** | OsRBCS2  (LOC_Os12g17600) | L601: 5′- AAGAAGGCGTACCCTGATGC -3′ | 192 bp | 2.052 +/- 0.017 | 0.998 |
|  |  | L602: 5′- ACGAAACAAGGTGGGAGACA -3′ |  |  |  |
|  | OsRBCS5  (LOC_Os12g19381) | L605: 5′- GGAAGCTGCCTATGTTCGGA -3′ | 200 bp | 2.039 +/- 0.014 | 0.999 |
|  |  | L606: 5′- GCTGGCTTGCCAACTAGCTTA -3′ |  |  |  |
|  | OsRBCS4  ([LOC_Os12g19470](http://rice.uga.edu/cgi-bin/ORF_infopage.cgi?orf=LOC_Os12g19470.2)) | L607: 5′- TACTTGCCGCCATTGACAGT -3′ | 182 bp | 2.040 +/- 0.009 | 0.999 |
|  |  | L608: 5′- GTGCATCCAAACATGGGCAG -3′ |  |  |  |
| **Jasmonate signaling pathway** | OsbHLH148  (LOC_Os03g53020) | L613: 5′- GGAAGCTGCCTATGTTCGGA -3′ | 90 bp | 2.034 +/- 0.051 | 0.983 |
|  |  | L614: 5′- GCTGGCTTGCCAACTAGCTTA -3′ |  |  |  |
|  | OsJAZ12  (LOC_Os10g25290) | L615: 5′- GGCCCAAACCCAAAGAGTTC -3′ | 198 bp | 2.231 +/- 0.038 | 0.994 |
|  |  | L616: 5′- GTGCATCCAAACATGGGCAG -3′ |  |  |  |
| **Oxidative-stress marker genes** | Cytochrome P450  (LOC_Os01g41810) | L629: 5′- TACGACTTTCGGCTTGTGCT -3′ | 220 bp | 2.028 +/- 0.051 | 0.984 |
|  |  | L630: 5′- TCTGAAAGCCCTCCTGGTCT -3′ |  |  |  |
| **Reference genes** | EIF5C  (LOC_Os11g21990.1) | Evd912 5′- CACGTTACGGTGACACCTTTT -3′ | 90 bp | 2.024 +/- 0.021 | 0.995 |
|  |  | Evd913: 5′- GACGCTCTCCTTCTTCCTCAG -3′ |  |  |  |
|  | EXP | Evd910: 5′- TGTGAGCAGCTTCTCGTTTG -3′ | 101 bp | 2.122 +/- 0.017 | 0.994 |
|  | (LOC_Os03g27010) | Evd911: 5′- TGTTGTTGCCTGTGAGATCG -3′ |  |  |  |
|  | EXPNarsai | L687: 5′- AGGAACATGGAGAAGAACAAGG -3′ | 112 bp | 1.981 +/- 0.014 | 0.997 |
|  | (LOC_Os07g02340.1) | L688: 5′- CAGAGGTGGTGCAGATGAAA -3′ |  |  |  |

**Table S2.** GO analysis of 199 annotated out of 215 down-regulated differentially expressed genes (DEGs) (log_2_FC<-1) unique in shoots of plants from line J at 3 h (MeJA-line J vs. Mock-line J)

| Identifier | Type | Log2-Enrichment Fold | P-Value | Subset Ratio | Description | Best Hierarchical | gene number |
| --- | --- | --- | --- | --- | --- | --- | --- |
| GO:0015995 | BP | 3.72 | 3.42E-02 | 3.18% | chlorophyll biosynthetic process | 1 | 5 |
| GO:0050794 | BP | 0.95 | 8.21E-03 | 26.75% | regulation of cellular process | 1 | 42 |
| GO:0050896 | BP | 0.77 | 7.24E-03 | 35.67% | response to stimulus | 1 | 56 |
| GO:0016607 | CC | 3.72 | 2.46E-02 | 2.55% | nuclear speck | 1 | 4 |
| GO:0005381 | MF | 5 | 2.66E-02 | 1.91% | iron ion transmembrane transporter activity | 1 | 3 |
| GO:0003700 | MF | 1.72 | 1.97E-02 | 8.92% | DNA-binding transcription factor activity | 1 | 14 |
| GO:0003677 | MF | 1.15 | 2.25E-02 | 15.92% | DNA binding | 1 | 27 |

**Table S3.** GO analysis of 358 annotated out of 377 down-regulated differentially expressed genes (DEGs) (log_2_FC < -1) unique in shoots of WT plants at 3 h (MeJA-WT vs. Mock-WT)

| Identifier | Type | Log2-Enrichment Fold | P-Value | Subset Ratio | Description | Best Hierarchical | gene number |
| --- | --- | --- | --- | --- | --- | --- | --- |
| GO:0006268 | BP | 6.17 | 1.46E-06 | 1.60% | DNA unwinding involved in DNA replication | 1 | 5 |
| GO:0006270 | BP | 5.01 | 2.00E-05 | 1.92% | DNA replication initiation | 1 | 6 |
| GO:0042793 | BP | 4.97 | 6.53E-03 | 1.28% | plastid transcription | 1 | 4 |
| GO:0070828 | BP | 4.73 | 1.38E-02 | 1.28% | heterochromatin organization | 1 | 4 |
| GO:0009658 | BP | 2.53 | 1.37E-03 | 3.85% | chloroplast organization | 1 | 12 |
| GO:0009657 | BP | 2.47 | 1.16E-04 | 4.81% | plastid organization | 1 | 15 |
| GO:0042555 | CC | 5.94 | 7.24E-07 | 1.60% | MCM complex | 1 | 5 |
| GO:0010369 | CC | 5.21 | 1.10E-06 | 1.92% | chromocenter | 1 | 6 |
| GO:0000229 | CC | 5.15 | 7.95E-08 | 2.24% | cytoplasmic chromosome | 1 | 7 |
| GO:0009508 | CC | 5.15 | 7.95E-08 | 2.24% | plastid chromosome | 1 | 7 |
| GO:0000347 | CC | 4.97 | 9.37E-04 | 1.28% | THO complex | 1 | 4 |
| GO:0000786 | CC | 4.8 | 1.07E-21 | 6.41% | nucleosome | 1 | 20 |
| GO:0042644 | CC | 4.16 | 3.54E-09 | 3.53% | chloroplast nucleoid | 1 | 11 |
| GO:0009505 | CC | 1.8 | 4.27E-03 | 4.81% | plant-type cell wall | 1 | 15 |
| GO:0009579 | CC | 1.73 | 1.37E-05 | 8.33% | thylakoid | 1 | 26 |
| GO:0009526 | CC | 1.34 | 1.19E-02 | 6.73% | plastid envelope | 1 | 21 |
| GO:0009507 | CC | 1.31 | 6.26E-12 | 24.36% | chloroplast | 1 | 76 |
| GO:0046982 | MF | 3.65 | 9.47E-15 | 6.73% | protein heterodimerization activity | 1 | 21 |

**Table S4.** GO analysis of 360 annotated out of 378 up-regulated differentially expressed genes (DEGs) (log_2_FC > 1) unique in shoots of WT plants at 3 h (MeJA-WT vs. Mock-WT)

| Identifier | Type | Log2-Enrichment Fold | P-Value | Subset Ratio | Description | Best Hierarchical | gene number |
| --- | --- | --- | --- | --- | --- | --- | --- |
| GO:0016143 | BP | 2.7 | 1.14E-02 | 2.83% | S-glycoside metabolic process | 1 | 9 |
| GO:0019757 | BP | 2.7 | 1.14E-02 | 2.83% | glycosinolate metabolic process | 1 | 9 |
| GO:0019760 | BP | 2.7 | 1.14E-02 | 2.83% | glucosinolate metabolic process | 1 | 9 |
| GO:0009414 | BP | 1.74 | 2.70E-04 | 7.55% | response to water deprivation | 1 | 24 |
| GO:0055114 | BP | 1.44 | 2.62E-08 | 16.67% | oxidation-reduction process | 1 | 53 |
| GO:0001101 | BP | 1.29 | 6.23E-06 | 15.09% | response to acid chemical | 1 | 48 |
| GO:0006970 | BP | 1.26 | 3.08E-02 | 8.18% | response to osmotic stress | 1 | 26 |
| GO:1901700 | BP | 1.23 | 1.52E-06 | 17.61% | response to oxygen-containing compound | 1 | 56 |
| GO:0009605 | BP | 1.07 | 2.40E-03 | 13.52% | response to external stimulus | 1 | 43 |
| GO:0010033 | BP | 1.03 | 1.93E-04 | 17.30% | response to organic substance | 1 | 55 |
| GO:0016705 | MF | 1.89 | 9.88E-05 | 6.60% | oxidoreductase activity, acting on paired donors, with incorporation or reduction of molecular oxygen | 1 | 21 |
| GO:0050662 | MF | 1.83 | 3.72E-04 | 6.29% | coenzyme binding | 1 | 22 |
| GO:0020037 | MF | 1.78 | 5.58E-05 | 7.55% | heme binding | 1 | 24 |
| GO:0005506 | MF | 1.75 | 2.53E-03 | 5.66% | iron ion binding | 1 | 19 |
| GO:0048037 | MF | 1.73 | 1.70E-10 | 14.78% | cofactor binding | 1 | 49 |
| GO:0016491 | MF | 1.53 | 2.13E-11 | 18.87% | oxidoreductase activity | 1 | 61 |

**Table S5.** GO analysis of 330 annotated out of 362 down-regulated differentially expressed genes (DEGs) (log_2_FC < -1) unique in shoots of plants from line J at 24 h (MeJA-line J vs. Mock-line J)

| Identifier | Type | Log2-Enrichment Fold | P-Value | Subset Ratio | Description | Best Hierarchical | gene number |
| --- | --- | --- | --- | --- | --- | --- | --- |
| GO:0110102 | BP | 5.66 | 2.00E-02 | 1.13% | chloroplast ribulose bisphosphate carboxylase complex assembly | 1 | 3 |
| GO:0015979 | BP | 2.4 | 4.97E-04 | 5.26% | photosynthesis | 1 | 14 |
| GO:0010598 | CC | 5.07 | 7.59E-04 | 1.50% | NAD(P)H dehydrogenase complex (plastoquinone) | 1 | 4 |
| GO:0009543 | CC | 3.66 | 7.56E-03 | 1.88% | chloroplast thylakoid lumen | 1 | 5 |
| GO:0031978 | CC | 3.66 | 7.56E-03 | 1.88% | plastid thylakoid lumen | 1 | 5 |
| GO:0031977 | CC | 3.59 | 5.40E-05 | 3.01% | thylakoid lumen | 1 | 8 |
| GO:0009579 | CC | 2.07 | 2.68E-08 | 10.53% | thylakoid | 1 | 28 |
| GO:0009507 | CC | 1.2 | 7.20E-08 | 22.56% | chloroplast | 1 | 60 |

**Table S6.** GO analysis of 437 annotated out of 472 down-regulated differentially expressed genes (DEGs) (log_2_FC < -1) unique in shoots of WT plants at 24 h (MeJA-WT vs. Mock-WT)

| Identifier | Type | Log2-Enrichment Fold | P-Value | Subset Ratio | Description | Best Hierarchical | gene number |
| --- | --- | --- | --- | --- | --- | --- | --- |
| GO:0045786 | BP | 3.56 | 1.41E-02 | 1.71% | negative regulation of cell cycle | 1 | 6 |
| GO:0051726 | BP | 2.5 | 2.71E-04 | 3.99% | regulation of cell cycle | 1 | 14 |
| GO:0044445 | CC | 2.11 | 2.01E-04 | 4.56% | cytosolic part | 1 | 16 |
| GO:0005694 | CC | 1.94 | 1.35E-02 | 3.42% | chromosome | 1 | 12 |
| GO:0048046 | CC | 1.51 | 1.85E-02 | 4.84% | apoplast | 1 | 17 |
| GO:0009532 | CC | 1.34 | 7.15E-04 | 8.26% | plastid stroma | 1 | 29 |
| GO:0044434 | CC | 1.29 | 1.49E-06 | 13.68% | chloroplast part | 1 | 48 |
| GO:0009507 | CC | 1.02 | 8.74E-07 | 19.94% | chloroplast | 1 | 70 |

**Table S7.** GO analysis of 320 annotated out of 338 up-regulated differentially expressed genes (DEGs) (log_2_FC > 1) unique in shoots of plants from line J at 24 h (MeJA-line J vs. Mock-line J)

| Identifier | Type | Log2-Enrichment Fold | P-Value | Subset Ratio | Description | Best Hierarchical | gene number |
| --- | --- | --- | --- | --- | --- | --- | --- |
| GO:0009751 | BP | 2.26 | 3.18E-03 | 4.96% | response to salicylic acid | 1 | 13 |
| GO:0009611 | BP | 2.01 | 4.51E-03 | 5.73% | response to wounding | 1 | 15 |
| GO:0046677 | BP | 1.99 | 2.60E-03 | 6.11% | response to antibiotic | 1 | 16 |
| GO:0009753 | BP | 1.91 | 2.06E-02 | 5.34% | response to jasmonic acid | 1 | 14 |
| GO:0042493 | BP | 1.83 | 6.97E-05 | 9.16% | response to drug | 1 | 24 |
| GO:1901700 | BP | 1.09 | 1.55E-03 | 16.03% | response to oxygen-containing compound | 1 | 42 |
| GO:0010033 | BP | 1.08 | 4.10E-04 | 17.94% | response to organic substance | 1 | 47 |
| GO:0005777 | CC | 2.08 | 1.67E-02 | 3.82% | peroxisome | 1 | 10 |
| GO:0042579 | CC | 2.08 | 1.67E-02 | 3.82% | microbody | 1 | 10 |

**Table S8.** GO analysis of 356 annotated out of 381 up-regulated differentially expressed genes (DEGs) (log_2_FC > 1) unique in shoots of WT plants at 24 h (MeJA-WT vs. Mock-WT)

| Identifier | Type | Log2-Enrichment Fold | P-Value | Subset Ratio | Description | Best Hierarchical | gene number |
| --- | --- | --- | --- | --- | --- | --- | --- |
| GO:0006527 | BP | 5.72 | 1.72E-02 | 0.98% | arginine catabolic process | 1 | 3 |
| GO:0006596 | BP | 4.39 | 3.78E-03 | 1.63% | polyamine biosynthetic process | 1 | 5 |
| GO:0009686 | BP | 4.09 | 1.38E-03 | 1.96% | gibberellin biosynthetic process | 1 | 6 |
| GO:0097164 | BP | 3.31 | 3.37E-02 | 1.96% | ammonium ion metabolic process | 1 | 6 |
| GO:0071229 | BP | 1.67 | 5.72E-03 | 6.54% | cellular response to acid chemical | 1 | 20 |
| GO:0042493 | BP | 1.41 | 4.09E-02 | 6.86% | response to drug | 1 | 21 |
| GO:0055085 | BP | 1.22 | 3.29E-02 | 8.82% | transmembrane transport | 1 | 29 |
| GO:0055114 | BP | 1.09 | 3.93E-03 | 13.07% | oxidation-reduction process | 1 | 40 |
| GO:1901700 | BP | 1.03 | 1.60E-03 | 15.36% | response to oxygen-containing compound | 1 | 47 |
| GO:0042221 | BP | 0.88 | 2.31E-04 | 22.22% | response to chemical | 1 | 68 |
| GO:0006950 | BP | 0.81 | 1.36E-03 | 22.22% | response to stress | 1 | 68 |
| GO:0000323 | CC | 4.72 | 2.22E-02 | 0.98% | lytic vacuole | 1 | 3 |
| GO:0009899 | MF | 5.29 | 8.44E-04 | 1.31% | ent-kaurene synthase activity | 1 | 4 |
| GO:0004373 | MF | 4.87 | 4.94E-02 | 0.98% | glycogen (starch) synthase activity | 1 | 3 |
| GO:0000287 | MF | 2.43 | 1.82E-02 | 2.94% | magnesium ion binding | 1 | 9 |
| GO:0022857 | MF | 1.23 | 5.42E-03 | 9.48% | transmembrane transporter activity | 1 | 30 |
| GO:0016491 | MF | 0.89 | 4.94E-02 | 12.09% | oxidoreductase activity | 1 | 37 |
| GO:0003824 | MF | 0.52 | 5.20E-06 | 50.65% | catalytic activity | 1 | 157 |

**Table S9.** GO analysis of 694 annotated out of 747 down-regulated differentially expressed genes (DEGs) (log_2_FC < -1) unique in roots of WT plants at 3 h (MeJA-WT vs. Mock-WT)

| Identifier | Type | Log2-Enrichment Fold | P-Value | Subset Ratio | Description | Best Hierarchical | gene number |
| --- | --- | --- | --- | --- | --- | --- | --- |
| GO:0010258 | BP | 5.21 | 1.77E-03 | 0.69% | NADH dehydrogenase complex (plastoquinone) assembly | 1 | 4 |
| GO:0019684 | BP | 2.5 | 8.46E-08 | 3.77% | photosynthesis, light reaction | 1 | 22 |
| GO:0009657 | BP | 2.36 | 1.53E-08 | 4.46% | plastid organization | 1 | 26 |
| GO:0015979 | BP | 2.21 | 6.40E-08 | 4.63% | photosynthesis | 1 | 27 |
| GO:0034641 | BP | 0.48 | 2.13E-02 | 22.47% | cellular nitrogen compound metabolic process | 1 | 135 |
| GO:0071704 | BP | 0.3 | 7.74E-03 | 45.11% | organic substance metabolic process | 1 | 272 |
| GO:0010598 | CC | 4.53 | 1.98E-05 | 1.03% | NAD(P)H dehydrogenase complex (plastoquinone) | 1 | 6 |
| GO:0009543 | CC | 3.21 | 4.32E-04 | 1.37% | chloroplast thylakoid lumen | 1 | 8 |
| GO:0031978 | CC | 3.21 | 4.32E-04 | 1.37% | plastid thylakoid lumen | 1 | 8 |
| GO:0042644 | CC | 2.97 | 4.12E-04 | 1.54% | chloroplast nucleoid | 1 | 9 |
| GO:0044436 | CC | 2.26 | 8.36E-18 | 8.58% | thylakoid part | 1 | 50 |
| GO:0031976 | CC | 2.23 | 1.49E-19 | 9.61% | plastid thylakoid | 1 | 56 |
| GO:0009579 | CC | 2.11 | 2.71E-20 | 10.81% | thylakoid | 1 | 63 |
| GO:0009941 | CC | 1.78 | 1.84E-12 | 8.92% | chloroplast envelope | 1 | 52 |
| GO:0009526 | CC | 1.77 | 1.38E-12 | 9.09% | plastid envelope | 1 | 53 |
| GO:0044434 | CC | 1.74 | 6.06E-27 | 18.70% | chloroplast part | 1 | 109 |
| GO:0008017 | MF | 2.15 | 1.75E-02 | 1.89% | microtubule binding | 1 | 11 |
| GO:0003723 | MF | 1 | 1.38E-02 | 6.52% | RNA binding | 1 | 41 |

**Table S10.** GO analysis of 449 annotated out of 477 up-regulated differentially expressed genes (DEGs) (log_2_FC > 1) unique in roots of WT plants at 3 h (MeJA-WT vs. Mock-WT)

| Identifier | Type | Log2-Enrichment Fold | P-Value | Subset Ratio | Description | Best Hierarchical | gene number |
| --- | --- | --- | --- | --- | --- | --- | --- |
| GO:0019218 | BP | 5.41 | 3.33E-02 | 0.79% | regulation of steroid metabolic process | 1 | 3 |
| GO:0010035 | BP | 0.97 | 4.18E-02 | 10.00% | response to inorganic substance | 1 | 38 |
| GO:1901700 | BP | 0.84 | 2.53E-02 | 13.42% | response to oxygen-containing compound | 1 | 51 |
| GO:0050662 | MF | 1.57 | 5.64E-03 | 5.26% | coenzyme binding | 1 | 22 |
| GO:0048037 | MF | 1.2 | 3.85E-04 | 10.26% | cofactor binding | 1 | 41 |
| GO:0003824 | MF | 0.44 | 5.41E-05 | 47.89% | catalytic activity | 1 | 189 |

**Table S11.** GO analysis of 521 annotated out of 587 up-regulated differentially expressed genes (DEGs) (log_2_FC > 1) unique in roots of plants from line J at 24 h (MeJA-line J vs. Mock-line J)

| Identifier | Type | Log2-Enrichment Fold | P-Value | Subset Ratio | Description | Best Hierarchical | gene number |
| --- | --- | --- | --- | --- | --- | --- | --- |
| GO:0046209 | BP | 5 | 5.42E-03 | 0.95% | nitric oxide metabolic process | 1 | 4 |
| GO:0042542 | BP | 2.82 | 1.99E-03 | 2.39% | response to hydrogen peroxide | 1 | 10 |
| GO:0009408 | BP | 2.06 | 2.16E-03 | 3.82% | response to heat | 1 | 16 |
| GO:0009415 | BP | 1.42 | 5.85E-03 | 6.21% | response to water | 1 | 26 |
| GO:0010035 | BP | 1.17 | 1.30E-04 | 11.46% | response to inorganic substance | 1 | 48 |
| GO:0009628 | BP | 0.99 | 4.74E-06 | 18.14% | response to abiotic stimulus | 1 | 76 |
| GO:1901700 | BP | 0.98 | 1.86E-04 | 14.80% | response to oxygen-containing compound | 1 | 62 |
| GO:0042221 | BP | 0.8 | 8.12E-05 | 21.00% | response to chemical | 1 | 88 |
| GO:0006950 | BP | 0.75 | 3.66E-04 | 21.24% | response to stress | 1 | 89 |
| GO:0050896 | BP | 0.66 | 2.17E-06 | 33.17% | response to stimulus | 1 | 139 |
| GO:0061630 | MF | 2.88 | 1.63E-02 | 1.67% | ubiquitin protein ligase activity | 1 | 7 |
| GO:0061659 | MF | 2.88 | 1.63E-02 | 1.67% | ubiquitin-like protein ligase activity | 1 | 7 |
| GO:0004842 | MF | 2.04 | 1.66E-04 | 4.30% | ubiquitin-protein transferase activity | 1 | 19 |

**Table S12.** GO analysis of 437 annotated out of 472 up-regulated differentially expressed genes (DEGs) (log_2_FC > 1) unique in roots of WT plants at 24 h (MeJA-WT vs. Mock-WT)

| Identifier | Type | Log2-Enrichment Fold | P-Value | Subset Ratio | Description | Best Hierarchical | gene number |
| --- | --- | --- | --- | --- | --- | --- | --- |
| GO:0006568 | BP | 3.78 | 3.61E-02 | 1.48% | tryptophan metabolic process | 1 | 5 |
| GO:0006586 | BP | 3.78 | 3.61E-02 | 1.48% | indolalkylamine metabolic process | 1 | 5 |
| GO:0042435 | BP | 3.32 | 7.88E-03 | 2.08% | indole-containing compound biosynthetic process | 1 | 7 |
| GO:0009073 | BP | 3.22 | 1.25E-02 | 2.08% | aromatic amino acid family biosynthetic process | 1 | 7 |
| GO:0009072 | BP | 3.04 | 4.95E-04 | 2.97% | aromatic amino acid family metabolic process | 1 | 10 |
| GO:0044283 | BP | 1.55 | 3.17E-03 | 7.12% | small molecule biosynthetic process | 1 | 25 |
| GO:0020037 | MF | 1.43 | 1.82E-02 | 5.93% | heme binding | 1 | 20 |
| GO:0098588 | CC | 1.14 | 1.57E-02 | 7.72% | bounding membrane of organelle | 1 | 26 |
| GO:0005794 | CC | 1.1 | 4.15E-02 | 7.12% | Golgi apparatus | 1 | 24 |
| GO:0012505 | CC | 0.92 | 5.88E-03 | 12.17% | endomembrane system | 1 | 41 |
| GO:0016740 | MF | 0.67 | 3.97E-03 | 22.55% | transferase activity | 1 | 79 |
| GO:0003824 | MF | 0.63 | 7.12E-11 | 54.60% | catalytic activity | 1 | 185 |
| GO:0016020 | CC | 0.48 | 5.75E-03 | 31.75% | membrane | 1 | 108 |

**Table S13.** GO analysis of 641 annotated out of 691 down-regulated differentially expressed genes (DEGs) (log_2_FC < -1) unique in roots of plants from line J at 24 h (MeJA-line J vs. Mock-line J)

| Identifier | Type | Log2-Enrichment Fold | P-Value | Subset Ratio | Description | Best Hierarchical | gene number |
| --- | --- | --- | --- | --- | --- | --- | --- |
| GO:0006364 | BP | 2.54 | 4.49E-03 | 2.03% | rRNA processing | 1 | 11 |
| GO:0042254 | BP | 2.37 | 1.39E-04 | 2.96% | ribosome biogenesis | 1 | 16 |
| GO:0034470 | BP | 2.22 | 4.74E-05 | 3.51% | ncRNA processing | 1 | 19 |
| GO:0051301 | BP | 1.66 | 4.12E-02 | 3.14% | cell division | 1 | 17 |
| GO:0006412 | BP | 1.35 | 2.74E-03 | 5.73% | translation | 1 | 31 |
| GO:0010467 | BP | 0.81 | 8.03E-07 | 21.44% | gene expression | 1 | 117 |
| GO:0034641 | BP | 0.8 | 9.40E-10 | 28.10% | cellular nitrogen compound metabolic process | 1 | 153 |
| GO:0046483 | BP | 0.7 | 1.73E-05 | 22.92% | heterocycle metabolic process | 1 | 125 |
| GO:1901360 | BP | 0.68 | 1.48E-05 | 24.03% | organic cyclic compound metabolic process | 1 | 131 |
| GO:0034645 | BP | 0.68 | 1.66E-03 | 17.74% | cellular macromolecule biosynthetic process | 1 | 96 |
| GO:1901576 | BP | 0.62 | 9.40E-05 | 24.77% | organic substance biosynthetic process | 1 | 134 |
| GO:0007275 | BP | 0.62 | 2.02E-02 | 16.27% | multicellular organism development | 1 | 88 |
| GO:0044249 | BP | 0.59 | 6.50E-04 | 23.66% | cellular biosynthetic process | 1 | 128 |
| GO:0071704 | BP | 0.44 | 2.03E-07 | 49.72% | organic substance metabolic process | 1 | 273 |
| GO:0044238 | BP | 0.41 | 1.72E-05 | 45.66% | primary metabolic process | 1 | 251 |

**Table S14.** GO analysis of 943 annotated out of 1017 down-regulated differentially expressed genes (DEGs) (log_2_FC < -1) unique in roots of WT plants at 24 h (MeJA-WT vs. Mock-WT)

| Identifier | Type | Log2-Enrichment Fold | P-Value | Subset Ratio | Description | Best Hierarchical | gene number |
| --- | --- | --- | --- | --- | --- | --- | --- |
| GO:0009658 | BP | 1.94 | 4.58E-04 | 2.55% | chloroplast organization | 1 | 20 |
| GO:0009657 | BP | 1.93 | 8.92E-06 | 3.32% | plastid organization | 1 | 26 |
| GO:0015979 | BP | 1.62 | 1.87E-03 | 3.07% | photosynthesis | 1 | 24 |
| GO:0009543 | CC | 3.36 | 2.41E-07 | 1.53% | chloroplast thylakoid lumen | 1 | 12 |
| GO:0031978 | CC | 3.36 | 2.41E-07 | 1.53% | plastid thylakoid lumen | 1 | 12 |
| GO:0031977 | CC | 3.04 | 1.68E-08 | 2.04% | thylakoid lumen | 1 | 16 |
| GO:0042644 | CC | 2.7 | 6.54E-04 | 1.28% | chloroplast nucleoid | 1 | 10 |
| GO:0009295 | CC | 2.63 | 3.29E-04 | 1.40% | nucleoid | 1 | 11 |
| GO:0042646 | CC | 2.63 | 3.29E-04 | 1.40% | plastid nucleoid | 1 | 11 |
| GO:0044436 | CC | 1.95 | 4.71E-15 | 6.90% | thylakoid part | 1 | 54 |
| GO:0031976 | CC | 1.9 | 3.85E-16 | 7.66% | plastid thylakoid | 1 | 60 |
| GO:0009570 | CC | 1.71 | 1.07E-18 | 10.34% | chloroplast stroma | 1 | 81 |
| GO:0044434 | CC | 1.63 | 1.80E-30 | 17.37% | chloroplast part | 1 | 136 |
| GO:0044435 | CC | 1.62 | 1.12E-30 | 17.62% | plastid part | 1 | 138 |
| GO:0009507 | CC | 1.41 | 7.38E-38 | 26.05% | chloroplast | 1 | 204 |
| GO:0048046 | CC | 1.12 | 1.43E-02 | 3.70% | apoplast | 1 | 29 |
| GO:0005576 | CC | 0.99 | 8.50E-03 | 4.85% | extracellular region | 1 | 38 |

**Table S15.** GO analysis of 35 common differentially expressed genes (DEGs) (log_2_FC>1) in root samples at both 3 h and 24h (Mock-line J vs. Mock-WT)

| Identifier | Type | Log2-Enrichment Fold | P-Value | Subset Ratio | Description | Best Hierarchical | gene number |
| --- | --- | --- | --- | --- | --- | --- | --- |
| GO:0032196 | BP | 7.38 | 1.23E-02 | 10.53% | transposition | 1 | 2 |
| GO:0016114 | BP | 5.13 | 1.55E-02 | 15.79% | terpenoid biosynthetic process | 1 | 3 |
| GO:0009686 | BP | 6.51 | 4.17E-02 | 10.53% | gibberellin biosynthetic process | 1 | 2 |
| GO:0010333 | MF | 5.71 | 3.93E-02 | 10.53% | terpene synthase activity | 1 | 2 |

**Table S16.** GO analysis of 35 common differentially expressed genes (DEGs) (log_2_FC>1) in shoot samples at both 3 h and 24h (Mock-line J vs. Mock-WT)

| Identifier | Type | Log2-Enrichment Fold | P-Value | Subset Ratio | Description | Best Hierarchical | gene number |
| --- | --- | --- | --- | --- | --- | --- | --- |
| GO:0032196 | BP | 7.31 | 1.51E-02 | 10.00% | transposition | 1 | 2 |
| GO:0034007 | MF | 10.39 | 4.39E-02 | 5.00% | S-linalool synthase activity | 1 | 1 |

**Table S17.** Differentially expressed genes (DEGs) involved in GO terms shown in Figure S11

| RAP | Id | Description |
| --- | --- | --- |
| Os02g0121700 | LOC_Os02g02930 | terpene synthase, putative, expressed |
| Os04g0178300 | LOC_Os04g09900 | ent-kaurene synthase, chloroplast precursor, putative, expressed |
| Os04g0179100 | LOC_Os04g10000 | sex determination protein tasselseed-2, putative, expressed |
| Os05g0438201 | LOC_Os05g36240 | expressed protein |
| Os07g0153150 | LOC_Os07g05840 | expressed protein |
| Os11g0475000 | LOC_Os11g28530 | terpene synthase, putative, expressed |

**Table S18.** 50 positively co-expressed genes of 40S ribosomal protein S5 (RPS5A) identified by Genevestigator on the mRNA-Seq platform

| Order | MSU Locus | Gene | Measure | Description |
| --- | --- | --- | --- | --- |
| 0 | LOC_Os11g29190 | LOC_Os11g29190 | LOC_Os11g29190 | 40S ribosomal protein S5, putative, expressed |
| 1 | LOC_Os12g07010 | LOC_Os12g07010 | LOC_Os12g07010 | ribosomal protein L3, putative, expressed |
| 2 | LOC_Os05g11710 | LOC_Os05g11710 | LOC_Os05g11710 | ribosomal protein L5, putative, expressed |
| 3 | LOC_Os03g08440 | LOC_Os03g08440 | LOC_Os03g08440 | ribosomal protein S2, putative, expressed |
| 4 | LOC_Os03g04750 | LOC_Os03g04750 | LOC_Os03g04750 | 60S ribosomal protein L21-2, putative, expressed |
| 5 | LOC_Os08g44480 | LOC_Os08g44480 | LOC_Os08g44480 | 40S ribosomal protein S25, putative, expressed |
| 6 | LOC_Os12g38000 | LOC_Os12g38000 | LOC_Os12g38000 | 60S ribosomal protein L8, putative, expressed |
| 7 | LOC_Os02g47140 | LOC_Os02g47140 | LOC_Os02g47140 | L11 domain containing ribosomal protein, putative, expressed |
| 8 | LOC_Os09g32976 | LOC_Os09g32976 | LOC_Os09g32976 | ribosomal protein L7Ae, putative, expressed |
| 9 | LOC_Os04g51630 | LOC_Os04g51630 | LOC_Os04g51630 | 60S ribosomal protein L7, putative, expressed |
| 10 | LOC_Os05g48310 | LOC_Os05g48310 | LOC_Os05g48310 | 60S ribosomal protein L35a-3, putative, expressed |
| 11 | LOC_Os08g02410 | LOC_Os08g02410 | LOC_Os08g02410 | 40S ribosomal protein S13, putative, expressed |
| 12 | LOC_Os01g49290 | LOC_Os01g49290 | LOC_Os01g49290 | WD repeat-containing protein, putative, expressed |
| 13 | LOC_Os01g09510 | LOC_Os01g09510 | LOC_Os01g09510 | 60S acidic ribosomal protein, putative, expressed |
| 14 | LOC_Os03g31090 | LOC_Os03g31090 | LOC_Os03g31090 | 40S ribosomal protein S19, putative, expressed |
| 15 | LOC_Os03g54890 | LOC_Os03g54890 | LOC_Os03g54890 | ribosomal protein L13, putative, expressed |
| 16 | LOC_Os02g37862 | LOC_Os02g37862 | LOC_Os02g37862 | 60S ribosomal protein L6, putative, expressed |
| 17 | LOC_Os11g06750 | LOC_Os11g06750 | LOC_Os11g06750 | ribosomal protein L3, putative, expressed |
| 18 | LOC_Os03g22180 | LOC_Os03g22180 | LOC_Os03g22180 | 60S ribosomal protein L18-3, putative, expressed |
| 19 | LOC_Os04g50990 | LOC_Os04g50990 | LOC_Os04g50990 | L11 domain containing ribosomal protein, putative, expressed |
| 20 | LOC_Os10g41470 | LOC_Os10g41470 | LOC_Os10g41470 | 60S ribosomal protein L27-3, putative, expressed |
| 21 | LOC_Os01g22490 | LOC_Os01g22490 | LOC_Os01g22490 | 40S ribosomal protein S27a, putative, expressed |
| 22 | LOC_Os03g58050 | LOC_Os03g58050 | LOC_Os03g58050 | ribosomal protein S13p/S18e, putative, expressed |
| 23 | LOC_Os10g08930 | LOC_Os10g08930 | LOC_Os10g08930 | S10/S20 domain containing ribosomal protein, putative, expressed |
| 24 | LOC_Os03g18570 | LOC_Os03g18570 | LOC_Os03g18570 | 40S ribosomal protein S7, putative, expressed |
| 25 | LOC_Os10g32820 | LOC_Os10g32820 | LOC_Os10g32820 | 60S ribosomal protein L21-2, putative, expressed |
| 26 | LOC_Os05g06770 | LOC_Os05g06770 | LOC_Os05g06770 | 40S ribosomal protein S27a, putative, expressed |
| 27 | LOC_Os02g01560 | LOC_Os02g01560 | LOC_Os02g01560 | 40S ribosomal protein S4, putative, expressed |
| 28 | LOC_Os02g21660 | LOC_Os02g21660 | LOC_Os02g21660 | L1P family of ribosomal proteins domain containing protein, expressed |
| 29 | LOC_Os03g63400 | LOC_Os03g63400 | LOC_Os03g63400 | transcription factor BTF3, putative, expressed |
| 30 | LOC_Os01g61814 | LOC_Os01g61814 | LOC_Os01g61814 | 40S ribosomal protein S23, putative, expressed |
| 31 | LOC_Os05g47890 | LOC_Os05g47890 | LOC_Os05g47890 | WD domain, G-beta repeat domain containing protein, expressed |
| 32 | LOC_Os05g38520 | LOC_Os05g38520 | LOC_Os05g38520 | 60S ribosomal protein L36-2, putative, expressed |
| 33 | LOC_Os03g59310 | LOC_Os03g59310 | LOC_Os03g59310 | ribosomal protein, putative, expressed |
| 34 | LOC_Os07g42450 | LOC_Os07g42450 | LOC_Os07g42450 | ribosomal protein S2, putative, expressed |
| 35 | LOC_Os04g28180 | LOC_Os04g28180 | LOC_Os04g28180 | ribosomal protein, putative, expressed |
| 36 | LOC_Os01g48770 | LOC_Os01g48770 | LOC_Os01g48770 | 60S ribosomal protein L37a, putative, expressed |
| 37 | LOC_Os05g41110 | LOC_Os05g41110 | LOC_Os05g41110 | ribosomal protein L7Ae, putative, expressed |
| 38 | LOC_Os01g01060 | LOC_Os01g01060 | LOC_Os01g01060 | 40S ribosomal protein S5, putative, expressed |
| 39 | LOC_Os07g08330 | LOC_Os07g08330 | LOC_Os07g08330 | ribosomal protein L4, putative, expressed |
| 40 | LOC_Os01g54870 | LOC_Os01g54870 | LOC_Os01g54870 | 60S ribosomal protein L18a, putative, expressed |
| 41 | LOC_Os05g48320 | LOC_Os05g48320 | LOC_Os05g48320 | 60S ribosomal protein L37a, putative, expressed |
| 42 | LOC_Os03g13170 | LOC_Os03g13170 | LOC_Os03g13170 | ubiquitin fusion protein, putative, expressed |
| 43 | LOC_Os09g31180 | LOC_Os09g31180 | LOC_Os09g31180 | ribosomal protein L6, putative, expressed |
| 44 | LOC_Os02g57540 | LOC_Os02g57540 | LOC_Os02g57540 | 60S ribosomal protein L28-1, putative, expressed |
| 45 | LOC_Os03g58204 | LOC_Os03g58204 | LOC_Os03g58204 | ribosomal protein L4, putative, expressed |
| 46 | LOC_Os03g10340 | LOC_Os03g10340 | LOC_Os03g10340 | 40S ribosomal protein S3a, putative, expressed |
| 47 | LOC_Os02g54470 | LOC_Os02g54470 | LOC_Os02g54470 | 60S ribosomal protein L35a-3, putative, expressed |
| 48 | LOC_Os12g03090 | LOC_Os12g03090 | LOC_Os12g03090 | ribosomal protein, putative, expressed |
| 49 | LOC_Os03g38260 | LOC_Os03g38260 | LOC_Os03g38260 | 60S ribosomal protein L19-3, putative, expressed |
| 50 | LOC_Os05g39960 | LOC_Os05g39960 | LOC_Os05g39960 | 40S ribosomal protein S26, putative, expressed |

**Table S19.** 6 out of top 50 positively co-expressed genes of RPS5A clustered in the biological process of response to cytokinin (GO:0009735)

|  |  | id | RAP | description |
| --- | --- | --- | --- | --- |
| LOC_Os01g01060 | coding | LOC_Os01g01060.MSUv7.0 | Os01g0100700 | 40S ribosomal protein S5, putative, expressed |
| LOC_Os02g37862 | coding | LOC_Os02g37862.MSUv7.0 | Os02g0591700 | 60S ribosomal protein L6, putative, expressed |
| LOC_Os02g57540 | coding | LOC_Os02g57540.MSUv7.0 | Os02g0821200 | 60S ribosomal protein L28-1, putative, expressed |
| LOC_Os03g58204 | coding | LOC_Os03g58204.MSUv7.0 | Os03g0796501 | ribosomal protein L4, putative, expressed |
| LOC_Os03g54890 | coding | LOC_Os03g54890.MSUv7.0 | Os03g0756000 | ribosomal protein L13, putative, expressed |
| LOC_Os07g08330 | coding | LOC_Os07g08330.MSUv7.0 | Os07g0180900 | ribosomal protein L4, putative, expressed |

**Table S20.** Expression levels of differentially expressed genes (DEGs) clustered in the biological process of response to cytokinin (GO:0009735) (MeJA vs. Mock)

| GO terms | Locus number | Annotation | 3 h shoot MeJA vs. Mock | | 24 h shoot MeJA vs. Mock | |
| --- | --- | --- | --- | --- | --- | --- |
|  |  |  | WT | J | WT | J |
|  |  |  | log2FoldChange (padj) | log2FoldChange (padj) | log2FoldChange (padj) | log2FoldChange (padj) |
| response to cytokinin (GO:0009735) | Os01g0100700 | 40S ribosomal protein S5, putative, expressed | N/A | N/A | -1.003247 (1.61E-08) | N/A |
|  | Os02g0591700 | 60S ribosomal protein L6, putative, expressed | N/A | N/A | -0.381484 (5.12E-04) | N/A |
|  | Os02g0821200 | 60S ribosomal protein L28-1, putative, expressed | N/A | N/A | N/A | N/A |
|  | Os03g0796501 | ribosomal protein L4, putative, expressed | N/A | N/A | -0.371393 (7.56E-04) | N/A |
|  | Os03g0756000 | ribosomal protein L13, putative, expressed | N/A | N/A | -0.625204 (3.43E-07) | N/A |
|  | Os07g0180900 | ribosomal protein L4, putative, expressed | N/A | N/A | -0.576799 (1.55E-07) | N/A |
|  | Os11g0482000 | 40S ribosomal protein S5, putative, expressed | N/A | N/A | -0.436728 (2.85E-04) | N/A |

**Table S21.** Genes involved in the gene regulatory network for OsRIP1 at 3 h (shown in Figure S11)

| Gene | RAP | Locus number | Description |
| --- | --- | --- | --- |
| OsRIP1 | Os01g0160800 | LOC_Os01g06740 | ribosome-inactivating protein, putative, expressed |
| CYP76M2 | Os08g0508000 | LOC_Os08g39730 | cytochrome P450, putative, expressed |
|  | Os02g0755900 | LOC_Os02g51930 | cytokinin-O-glucosyltransferase 2, putative, expressed |
| ICL | Os07g0529000 | LOC_Os07g34520 | isocitrate lyase, putative, expressed |
| UCL25 | Os08g0137900 | LOC_Os08g04350 | plastocyanin-like domain containing protein, putative, expressed |
| PR4C | Os11g0592000 | LOC_Os11g37950 | WIP3 - Wound-induced protein precursor, expressed |
| Os04g0344100 | Os04g0344100 | LOC_Os04g27670 | terpene synthase family, metal binding domain containing protein, expressed |

**Table S22.** Differentially expressed genes (DEGs) (log_2_FC > 2) unique in shoots of plants from line J at 24 h (MeJA-line J vs. Mock-line J) clustered in the molecular function of antioxidant activity (GO:0016209)

| Locus number | RAP | description |
| --- | --- | --- |
| LOC_Os05g04490.MSUv7.0 | Os05g0135400 | peroxidase precursor, putative, expressed |
| LOC_Os06g32990.MSUv7.0 | Os06g0521500 | peroxidase precursor, putative, expressed |
| LOC_Os07g44440.MSUv7.0 | Os07g0638400 | peroxiredoxin, putative, expressed |
| LOC_Os07g48030.MSUv7.0 | Os07g0677300 | peroxidase precursor, putative, expressed |
| LOC_Os08g35210.MSUv7.0 | Os08g0453766 | ferric reductase, putative, expressed |
| LOC_Os12g26290.MSUv7.0 | Os12g0448900 | alpha-DOX2, putative, expressed |
